# IS*1*-mediated chromosomal amplification of the *arn* operon leads to polymyxin B resistance in *Escherichia coli* B strains

**DOI:** 10.1128/mbio.00634-24

**Published:** 2024-06-21

**Authors:** Michael Maybin, Aditi M. Ranade, Ursula Schombel, Nicolas Gisch, Uwe Mamat, Timothy C. Meredith

**Affiliations:** 1 Department of Biochemistry and Molecular Biology, The Pennsylvania State University, University Park, Pennsylvania, USA; 2 The Huck Institutes of the Life Sciences, The Pennsylvania State University, University Park, Pennsylvania, USA; 3 Division of Bioanalytical Chemistry, Priority Research Area Infections, Research Center Borstel, Leibniz Lung Center, Borstel, Germany; 4 Division of Cellular Microbiology, Priority Research Area Infections, Research Center Borstel, Leibniz Lung Center, Leibniz Research Alliance INFECTIONS, Borstel, Germany; Yale School of Medicine, New Haven, Connecticut, USA; Emory University Vaccine Center, Atlanta, Georgia, USA

**Keywords:** lipopolysaccharide, polymyxins, chromosomal amplification, *arn* operon, *Escherichia coli*, insertion sequence elements, heteroresistance, antimicrobial resistance

## Abstract

Polymyxins [colistin and polymyxin B (PMB)] comprise an important class of natural product lipopeptide antibiotics used to treat multidrug-resistant Gram-negative bacterial infections. These positively charged lipopeptides interact with lipopolysaccharide (LPS) located in the outer membrane and disrupt the permeability barrier, leading to increased uptake and bacterial cell death. Many bacteria counter polymyxins by upregulating genes involved in the biosynthesis and transfer of amine-containing moieties to increase positively charged residues on LPS. Although 4-deoxy-l-aminoarabinose (Ara4N) and phosphoethanolamine (PEtN) are highly conserved LPS modifications in *Escherichia coli*, different lineages exhibit variable PMB susceptibilities and frequencies of resistance for reasons that are poorly understood. Herein, we describe a mechanism prevalent in *E. coli* B strains that depends on specific insertion sequence 1 (IS*1*) elements that flank genes involved in the biosynthesis and transfer of Ara4N to LPS. Spontaneous and transient chromosomal amplifications mediated by IS*1* raise the frequency of PMB resistance by 10- to 100-fold in comparison to strains where a single IS*1* element located 90 kb away from the end of the *arn* operon has been deleted. Amplification involving IS*1* becomes the dominant resistance mechanism in the absence of PEtN modification. Isolates with amplified *arn* operons gradually lose their PMB-resistant phenotype with passaging, consistent with classical PMB heteroresistance behavior. Analysis of the whole genome transcriptome profile showed altered expression of genes residing both within and outside of the duplicated chromosomal segment, suggesting complex phenotypes including PMB resistance can result from tandem amplification events.

**IMPORTANCE:**

Phenotypic variation in susceptibility and the emergence of resistant subpopulations are major challenges to the clinical use of polymyxins. While a large database of genes and alleles that can confer polymyxin resistance has been compiled, this report demonstrates that the chromosomal insertion sequence (IS) content and distribution warrant consideration as well. Amplification of large chromosomal segments containing the *arn* operon by IS*1* increases the Ara4N content of the lipopolysaccharide layer in *Escherichia coli* B lineages using a mechanism that is orthogonal to transcriptional upregulation through two-component regulatory systems. Altogether, our work highlights the importance of IS elements in modulating gene expression and generating diverse subpopulations that can contribute to phenotypic polymyxin B heteroresistance.

## INTRODUCTION

Antimicrobial resistance (AMR) is of increasing global concern as a significant contributor to deaths involving bacterial infections. In 2019, approximately 25% of the 5 million bacterial infection deaths were directly associated with AMR (1). As AMR is predicted to continually increase in the coming decades (2), efforts to develop new antibiotics as well as to improve the efficacy of current antibiotics by limiting resistance are critical. The polymyxin family of natural product antibiotics was first isolated in the 1940s from *Paenibacillus polymyxa* extracts (3), but challenging pharmacodynamics and nephrotoxicity limited the use of the two most highly active congeners [polymyxin B (PMB) and polymyxin E (colistin)] to animal agriculture (4, 5) and topical applications (6). The polymyxin family of lipopeptide antibiotics, however, has reemerged in the clinic (7–10), particularly for treating carbapenem-resistant Gram-negative bacterial infections involving *Enterobacteriaceae*, multidrug-resistant (MDR) *Pseudomonas aeruginosa*, and MDR *Acinetobacter baumannii* strains (11).

Polymyxins have an acylated tripeptide stem connected to a cyclic heptapeptide with several cationic amino acid side chains (12–14). Ionic interactions between negatively charged lipid A phosphate groups of lipopolysaccharide (LPS) molecules embedded in the outer membrane enhance cell envelope permeability, increasing access to LPS within the inner cytoplasmic membrane that is in transit which in turn ultimately leads to cell death (15). A common polymyxin resistance mechanism in *Escherichia coli* and other closely related *Enterobacteriaceae* involves enhanced masking of LPS phosphate groups on lipid A with positively charged residues (4, 5, 16–20), including 4-deoxy-l-aminoarabinose (Ara4N, encoded by *arn/pmr* genes) and phosphoethanolamine (PEtN, encoded by *ept/pmr* genes) groups. The levels of Ara4N/PEtN on LPS are controlled by a complex, dynamically regulated signaling network that integrates environmental growth cues, including concentrations of metal ions, pH, presence of cationic peptides, and general outer membrane fluidity (21). Mutations either in the two-component transcriptional regulatory systems themselves [PmrAB (13, 14) and PhoPQ (15)], in cognate sRNA-negative regulators [MgrR (22), MicA (23)], or in small accessory membrane proteins [MgrB (24)] all impart polymyxin resistance by upregulating *arn*/*ept* gene expression. Polymyxin resistance can also be acquired by horizontal gene transfer, most notably involving plasmids and transposons carrying *mcr* (mobile colistin resistance) genes encoding lipid A-PEtN transferases (25, 26). More recently, targeted proteolysis of Mcr by cell envelope stress response systems (20) and mutations in primary metabolic pathways contributing to the Ara4N/PEtN precursor pool (27) have also been shown to regulate LPS modification levels and phenotypic polymyxin resistance.

In comparison to stably inherited genetic mutations and plasmid-borne polymyxin resistance determinants, polymyxin heteroresistance (HR) is less well understood (28–30). HR refers to a resistant subpopulation, within a larger susceptible population, that demonstrates phenotypic resistance at eight times the typical MIC with an unstably inherited phenotype (31, 32). MIC microdilution susceptibility strain testing with polymyxins is often plagued by irreproducibility and the phenomena of “well-skipping,” whereby growth occasionally occurs at drug concentrations above the apparent MIC (19, 33–37). Resistance at elevated polymyxin concentrations, however, is not necessarily retained upon subculture and has further complicated the establishment of therapeutic susceptibility breakpoints (38, 39). Herein, we present evidence for one mechanism of polymyxin HR involving insertion sequence (IS) elements in *E. coli* B, which unlike *E. coli* K-12 strains can appear phenotypically resistant to polymyxins (40) and has high rates of HR. IS elements are a large class of small, integrated DNA elements flanked by inverted terminal repeats that encode a transposase for self-transposition (41–43). We show that a single insertion sequence (IS*1*-18) cassette located ~90 kb downstream from the *arn* operon acts in concert with copies of upstream IS*1* elements to drive tandem chromosomal amplification events. Amplified mutants have increased *arn* operon transcript levels resulting in higher amounts of Ara4N-modified LPS that in turn confers polymyxin resistance. Chromosomal amplification of the *arn* operon occurs widely across various wild-type *E. coli* B lineages that share an *arn* operon flanked by IS*1* elements and becomes the dominant route for polymyxin resistance when PEtN modification of lipid A is limited.

## RESULTS

### *E. coli* B PMB heteroresistance depends on the *arn* operon

**Fig 1:**
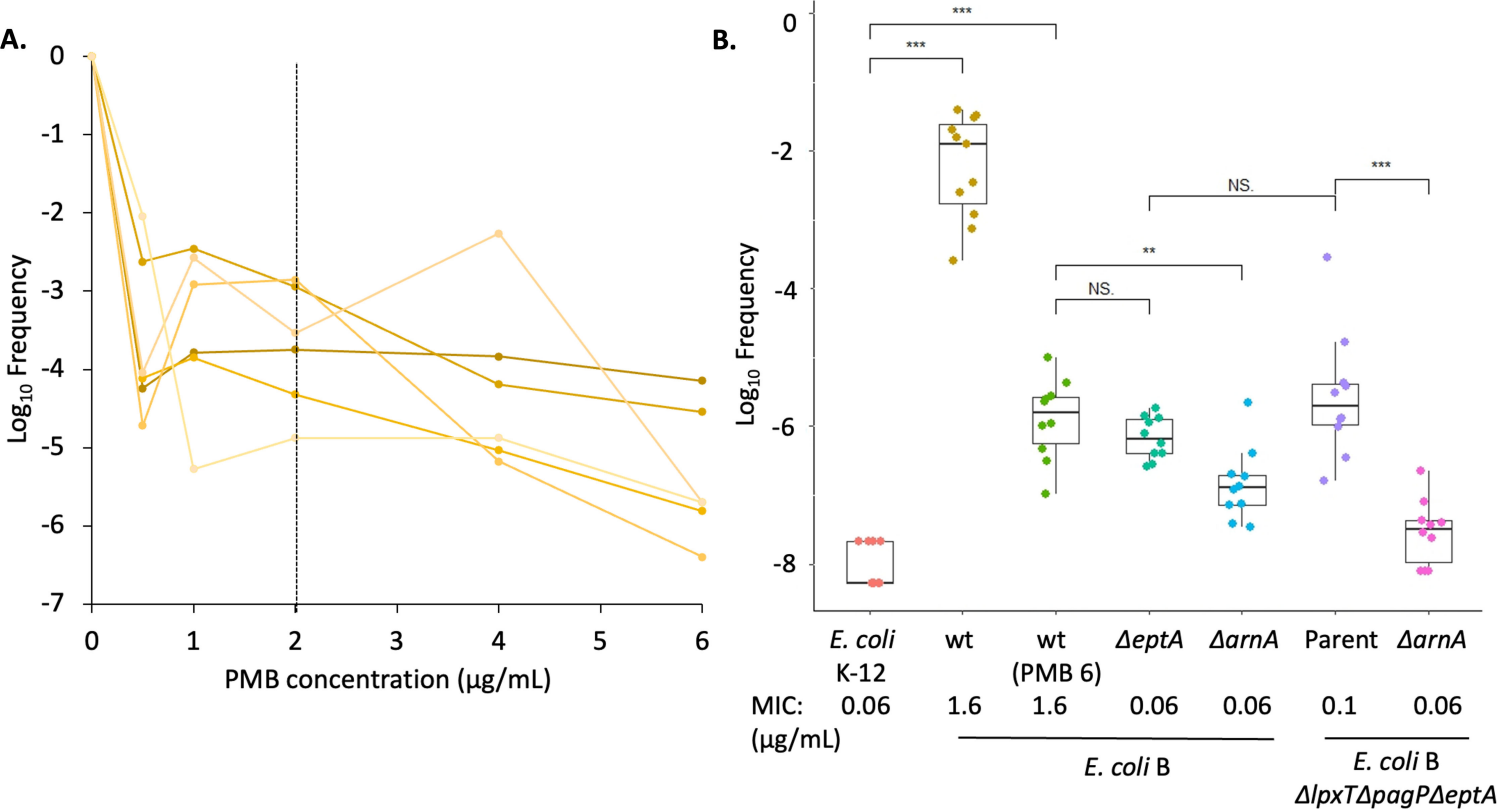
Heteroresistance leads to inconsistency between MIC and frequency of resistance. (**A**) Early exponential phase cells of *E. coli* BL21(DE3) were harvested, washed, diluted, and plated on lysogeny broth agar (LBA) with 0, 0.5, 1, 2, 4, and 6 µg/mL PMB. Log_10_ frequency of resistance was plotted against PMB concentration to generate the PAP. Each line represents a biological replicate (*n* = 6). The dotted line represents the USCAST breakpoint value of PMB. (**B**) Frequency of resistance was determined for 10 separate cultures per strain by plating dilutions of exponentially growing cells [optical density (OD_600_) of 0.2 to 0.7] on LBA containing 1 µg/mL of PMB [6 µg/mL PMB for wild-type (wt) PMB6] and then counting the ratio of resistant CFU to total plated cells after overnight incubation. Strain MIC values are based on the mean of three biological replicates. Significance values based on Student’s *t*-test (**P < 0.01 and ***P < 0.001; NS, not significant).

There are numerous conflicting reports on whether *E. coli* B strains are sensitive to polymyxins [reviewed by Schumann et al. (40)]. We also observed highly variable MIC values and well skipping in the *E. coli* B background, with MICs (0.06 to 4 μg/mL, mean 1.6 μg/mL) suffering from poor reproducibility (Table S1). To address whether HR may contribute to phenotypic variability, the *E. coli* BL21(DE3) wild type was tested using a population analysis profile (PAP) (Fig. 1A). While the bulk of the population was susceptible to low PMB concentrations (between 0.5 and 1 μg/mL), a substantial preexisting subpopulation was resistant to PMB at concentrations much greater than the USCAST-recommended breakpoint of 2 μg/mL for *Enterobacteriaceae* (Fig. 1A) (39). In contrast, MIC values for the *E. coli* K-12 strain BW25113 were consistently at 0.06 μg/mL with no well skipping. Deletion of either *eptA* or *arnA* in the *E. coli* B strain resulted in MICs in line with the K-12 strain (Fig. 1B; Table S1). However, the frequency of resistance (FOR) only in the Δ*arnA* background was at least 10-fold lower despite near identical MICs with Δ*eptA* (Fig. 1B). To further isolate a potential role of the *arn* operon in PMB HR, we constructed an *E. coli* B Δ*eptA* mutant that also lacked *lpxT* and *pagP*. LpxT adds a phosphate to the 1-position of lipid A that increases the negative charge while preventing further modification (44, 45), whereas PagP appends an acyl chain to form hepta-acylated lipid A (46). Either modification can alter susceptibility to cationic peptides by competing with ArnT for lipid A substrate (*lpxT*) or altering outer membrane permeability (*pagP*). In this strain background (Δ*eptA*Δ*lpxT*Δ*pagP*), referred to herein as the Parent strain, similar MICs and FOR rates to the Δ*eptA* strain indicate LpxT and PagP are minor factors in PMB resistance. Deleting *arnA* in the Parent strain once again decreased the FOR (Fig. 1B), consistent with a central role of the *arn* operon in PMB HR.

### PMB-resistant *E. coli* B isolates increase modification of lipid A with Ara4N

**Fig 2:**
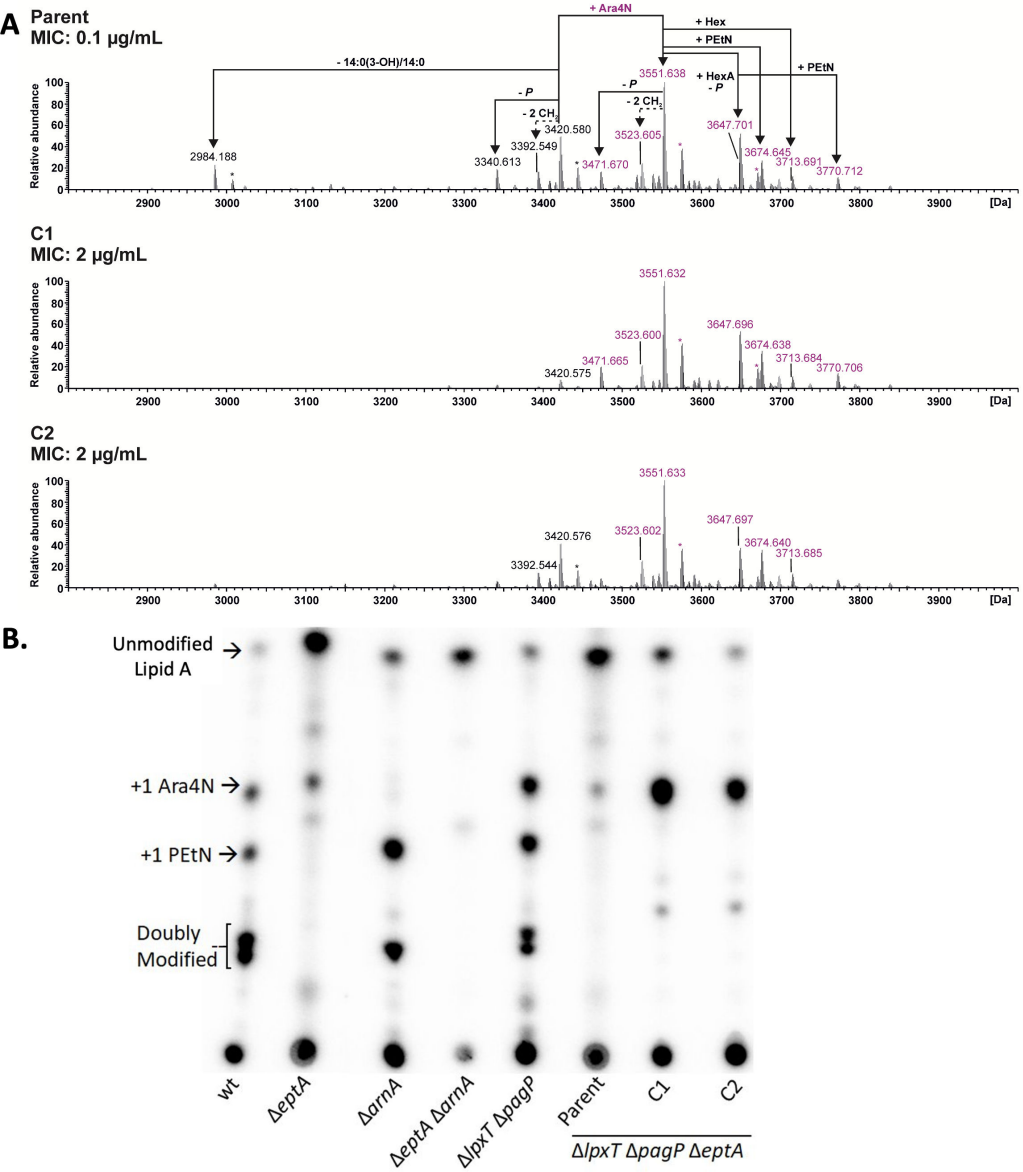
Polymyxin B-resistant isolates C1/C2 have increased relative levels of Ara4N-modified LPS. (**A**) ESI-MS spectra of LPS isolated from the PMB-sensitive Parent strain (top) and PMB-resistant isolates C1 (middle) and C2 (bottom). Charge-deconvoluted spectra of the MS analyses performed in negative ion mode are depicted (section: 2,800–4,000 Da), with all Ara4N-containing LPS species labeled in pink and all non-Ara4N-containing LPS species in *black*. Non-stoichiometric substitutions of the oligosaccharide core with PEtN, hexose (Hex), hexuronic acid (hexA), and phosphate (P) are indicated. Variations in total acyl chain length (−2 CH_2_) and sodium ion adducts (*) are indicated. Calculated monoisotopic masses for observed LPS species are summarized in Table 1. (**B**) TLC analysis of ^32^P-labeled lipid A isolated from *E. coli* BL21(DE3) wt, Δ*eptA*, Δ*arnA*, Δ*eptA*Δ*arnA*, Δ*lpxT*Δ*pagP,* Parent, and C1/C2 PMB-resistant isolates. Lipid A structures were assigned based on migration similarity to control strains.

Our prior work using electrospray ionization mass spectrometry (ESI-MS) showed elevated levels of both PEtN- and Ara4N-containing LPS in wild-type *E. coli* BL21(DE3) (47, 48), though total levels remain subsaturating. To determine if the PMB-resistant subpopulation had increased Ara4N levels, individual colonies were isolated from the Parent strain. Of the 26 non-clonal PMB-resistant strains isolated from independent cultures, all 26 displayed stable low-level PMB resistance well above the Parent strain MIC (2–4 versus 0.1 μg/mL). Two isolates (C1 and C2) were taken forward for LPS purification and analysis (Fig. 2A). SDS-PAGE separation of extracted LPS did not reveal major changes in either the total yield or banding profiles (Fig. S1). ESI-MS analyses of these LPS preparations, however, showed a marked relative shift from a near-equal distribution of unmodified and Ara4N-modified LPS chemotypes in the Parent strain to a majority containing Ara4N in C1 and C2 (Fig. 2A; Table 1). Whereas the MS spectra for LPS from the Parent strain and C1 were similar among two biological replicates, the content of hexuronic acid (HexA)- and PEtN-containing molecules for C2 LPS was variable (Fig. 2A; Fig. S2; Data File S1). To verify the additional Ara4N was attached to lipid A, LPS was labeled with inorganic ^32^P, hydrolyzed, and the released lipid A analyzed by thin layer chromatography (TLC). Both the C1 and C2 mutants contained more Ara4N-modified lipid A in comparison to the Parent (Fig. 2B), qualitatively consistent with ESI-MS peak area analysis of purified LPS (see Date File S1). Furthermore, analysis of the core oligosaccharide released after mild acidic hydrolysis of purified LPS did not detect any Ara4N substituents (Fig. S3), while singly and doubly Ara4N-modified lipid A was readily detected in C1 and C2 (Fig. S4). A similar shift toward enriched lipid A-Ara4N chemotype content was also readily apparent in the majority of 24 other independently isolated PMB-resistant mutants (Fig. S5), suggesting a common PMB resistance mechanism that leads to higher amounts of Ara4N-modified lipid A.

**TABLE 1:** Mass spectrometric analysis of LPS from PMB-sensitive Parent strain and PMB-resistant isolates C1 and C2^*c*^

Calc. mass (Da)	Composition	Parent		C1		C2	
		Obs. mass (Da)	Error (ppm)	Obs. mass (Da)	Error (ppm)	Obs. mass (Da)	Error (ppm)
2,984.181	LA_tetra_^*b*^ + Kdo_2_Hep_3_Hex_2_P_2_PEtN_1_	2,984.188	2.3	n.d.	–	2,984.187	2.0
3,340.607	LA_hexa_^*a*^ + Kdo_2_Hep_3_Hex_2_P_1_PEtN_1_	3,340.613	1.8	3,340.610	0.9	3,340.610	0.9
3,392.542	LA_hexa_ + Kdo_2_Hep_3_Hex_2_P_2_PEtN_1_*- 2 CH_2_*	3,392.549	2.1	3,392.543	0.3	3,392.544	0.6
3,420.573	LA_hexa_ + Kdo_2_Hep_3_Hex_2_P_2_PEtN_1_	3,420.580	2.0	3,420.575	0.6	3,420.576	0.9
**3,471.665**	LA_hexa_ + Kdo_2_Hep_3_Hex_2_**Ara4N_1_**P_1_PEtN_1_	3,471.670	1.4	3,471.665	0.0	3,471.666	0.3
3,516.639	LA_hexa_ + Kdo_2_Hep_3_Hex_2_HexA_1_P_1_PEtN_1_	3,516.643	1.1	3,516.638	−0.3	3,516.641	0.6
**3,523.600**	LA_hexa_ + Kdo_2_Hep_3_Hex_2_**Ara4N_1_**P_2_PEtN_1_ *- 2 CH_2_*	3,523.605	1.4	3,523.600	0.0	3,523.602	0.6
**3,537.616**	LA_hexa_ + Kdo_2_Hep_3_Hex_2_**Ara4N_1_**P_2_PEtN_1_ *- CH_2_*	3,537.621	1.4	3,537.615	−0.3	3,537.617	0.3
**3,551.631**	LA_hexa_ + Kdo_2_Hep_3_Hex_2_**Ara4N_1_**P_2_PEtN_1_	3,551.638	2.0	3,551.632	0.3	3,551.633	0.6
**3,647.697**	LA_hexa_ + Kdo_2_Hep_3_Hex_2_HexA_1_**Ara4N_1_**P_1_PEtN_1_	3,647.701	1.1	3,647.696	−0.3	3,647.697	0.0
**3,674.640**	LA_hexa_ + Kdo_2_Hep_3_Hex_2_**Ara4N_1_**P_2_PEtN_2_	3,674.645	1.4	3,674.638	−0.5	3,674.640	0.0
**3,713.684**	LA_hexa_ + Kdo_2_Hep_3_Hex_3_**Ara4N_1_**P_2_PEtN_1_	3,713.691	1.9	3,713.684	0.0	3,713.685	0.3
**3,770.706**	LA_hexa_ + Kdo_2_Hep_3_Hex_2_HexA_1_**Ara4N_1_**P_1_PEtN_2_	3,770.712	1.6	3,770.706	0.0	3,770.706	0.0

^*a*^ LA_hexa_ = lipid A, hexa-acylated: 2*GlcN, 2 P, 2*14:0(3-OH), 1*14:0[3-O(12:0)], 1*14:0[3-O(14:0)].

^*b*^ LA_tetra_ = lipid A, tetra-acylated: 2*GlcN, 2 P, 2*14:0(3-OH), 1*14:0[3-O(12:0)].

^*c*^ Summary of calculated monoisotopic neutral masses and observed molecular masses (Da), the corresponding MS spectra are depicted in Fig. 2A. Only LPS molecules for which the relative intensity of the first isotopic peak was above 10% compared with the first isotopic peak of the most abundant LPS molecule in at least one of the spectra were included. Salt adduct ions are not listed (a more detailed list can be found in Data File S1). Ara4N-containing molecules are highlighted in bold, and the accuracy of the measurement is stated as Δppm.

### Insertion sequences flank chromosomal amplifications containing the *arn* operon and impart transient PMB resistance

**Fig 3:**
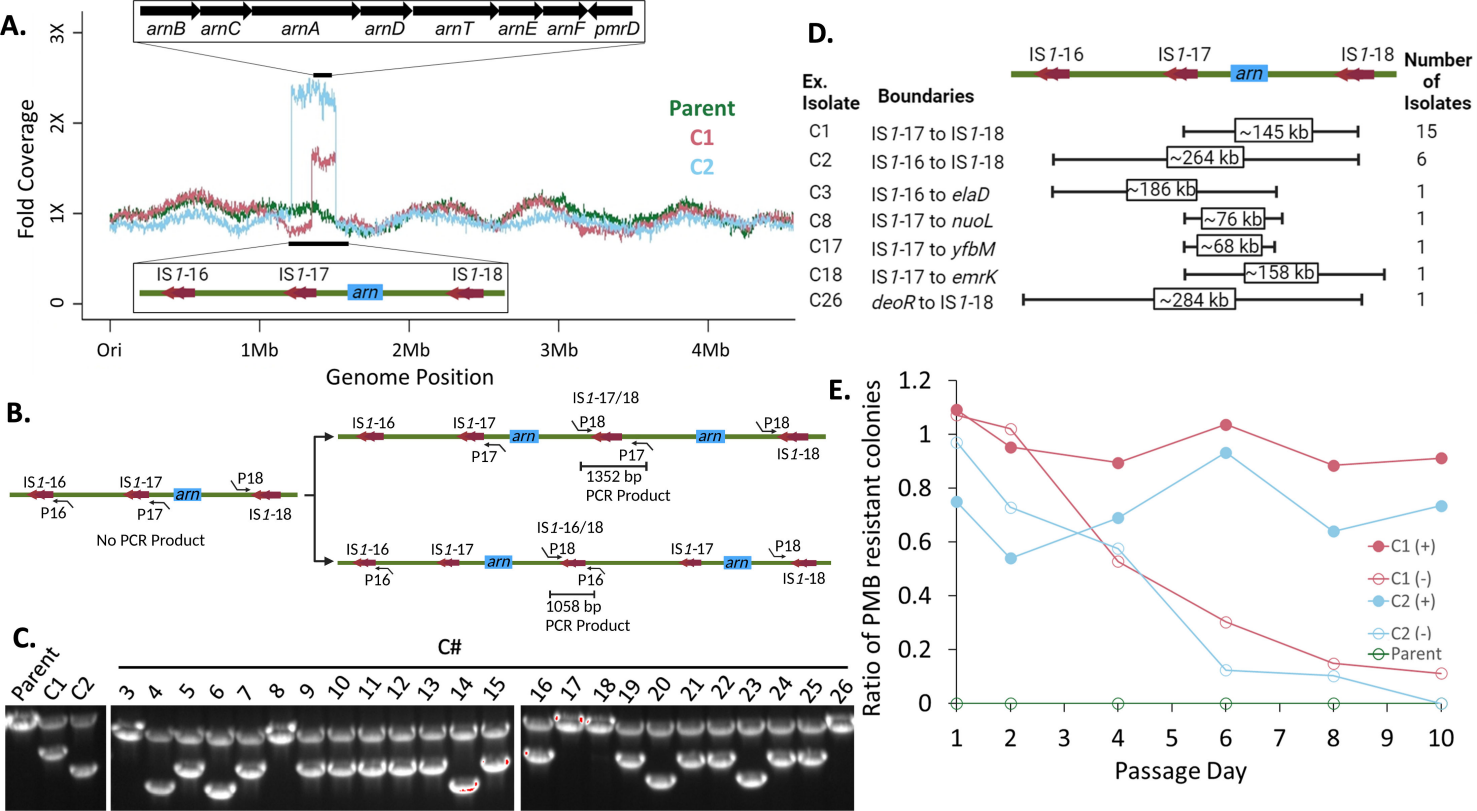
Insertion sequences flank chromosomal amplifications in PMB-resistant isolates. (A) The fold coverage was obtained by normalizing the position-specific read count to the mean read count across the entire chromosome, excluding the amplified regions for C1 (pink) and C2 (light blue). Parent fold coverage (dark green) obtained by normalizing to the mean read count across the entire chromosome. Amplified region of chromosomal DNA is highlighted with the flanking IS elements indicated (bottom inset). The *arn* operon and its location in the genome are indicated (top inset). (B) Diagram of the junctions formed via tandem amplifications involving IS*1*−17/IS*1*-18 (C1-like, top) and IS*1*−16/IS*1*-18 (C2-like, bottom). Relative locations of the IS*1* elements, *arn* operon, and primers used for multiplex junction PCR are indicated. (C) Multiplex junction PCR for the Parent strain and the PMB-resistant C1/C2 mutants used to confirm head-to-tail amplification (left subpanel). Primers for *pykA* were included as an internal control for amplification (2,116 bp), while they could be separated from IS*1*-16/IS*1*-18 (1,058 bp) and IS*1*-17/IS*1*-18 (1,352 bp) junction amplicons. An additional set of 24 independently isolated PMB-resistant colonies was likewise analyzed (right subpanel). (**D**) Diagram of the amplified regions among the 26 isolates. Boundaries of the C1 and C2 mutants are displayed as well as the boundaries of the five isolates bounded by only one IS element that failed to yield a diagnostic PCR product and were determined by whole genome sequencing. (**E**). IS-flanked amplifications in mutants are unstable and result in a transient PMB-resistant phenotype. Ratio of PMB-resistant colonies in Parent (green open circles) and tandemly amplified isolates (pink circles, C1, and blue circles, C2) over 10 days of passaging in the presence (solid circles) or absence (open circles) of 1 µg/mL PMB (data shown are representative of three independent biological replicates).

Polymyxin resistance via activating point mutations in the *arn/ept* operon regulatory network that produced highly modified LPS is commonly encountered, both in *in vitro* (19, 49, 50) and in clinical isolates (51, 52). However, targeted sequencing of genes associated with PMB resistance for resistant alleles did not reveal any candidates. We therefore used short-read Illumina next-generation sequencing to identify mutations in C1 and C2 (Fig. 3A). In both PMB-resistant isolates, plateaus of higher relative read coverage encompassing the *arn* operon were observed. Coverage increased nearly two- and threefold in C1 and C2, respectively, in comparison to the Parent strain. Plateau regions were bounded on both sides by IS*1* elements (Fig. 3A), a 768-bp mobile genetic element that is present in multiple copies [29 in *E. coli* BL21(DE3) (53)]. IS*1*-17 and IS*1*-18 flanked the C1-amplified region, whereas IS*1*-16 and IS*1*-18 flanked the C2-amplified region. The three IS*1* element alleles involved are nearly identical, save for a single unique bp in each. DNA amplification events bounded by repetitive stretches of DNA are thought to arise from an unequal DNA exchange between sister chromosomes resulting in head-to-tail duplication with an intervening third copy of the repeated element (Fig. 3B) (54–56). Multiplex PCR using five primers (two reverse primers annealing downstream of IS*1*-16 or IS*1*-17 and a forward primer annealing upstream of IS*1*-18 along with a primer pair to amplify the *pykA* control locus residing outside of plateau region) was thus used to confirm novel junction points (Fig. 3B). PCR products consistent in size with amplification events bounded by IS*1*-17/IS*1*-18 for C1 and IS*1*-16/IS*1*-18 for C2 were only observed in the C1 and C2 PMB-resistant mutants and not the Parent strain (Fig. 3C). Sanger sequencing of the novel, hybrid IS*1* junction PCR products confirmed duplication as diagrammed in Fig. 3B, with crossovers retaining the IS*1*-17 allele in C1 and the IS*1*-18 allele in C2. Likewise, 19 out of 24 of the other PMB-resistant isolates yielded PCR products with analogous amplification boundaries to C1 or C2 (Fig. 3C). The remaining five isolates that failed to produce junction PCR products were whole genome sequenced. While once again, all had increased read coverage encompassing the *arn* operon, only a single boundary involved one of the above IS*1* elements (Fig. 3D). These isolates either represent minor amplification pathways or more likely underwent further rearrangement through recombination events which is often observed with long segments of tandemly amplified DNA (55–57). In either case, all isolates acquired PMB resistance through *arn* operon amplification mediated by flanking IS*1* elements.

To determine the stability of the amplifications, we performed outcrossing experiments where the Parent, C1, and C2 isolates were passaged in the presence or absence of PMB over 10 days. Aliquots were plated on LBA plates with and without PMB. In the absence of PMB, the population of PMB susceptible cells increased, whereas mutants passaged in the presence of PMB largely retained the resistant phenotype (Fig. 3E). PMB resistance was also unstable to varying extents among the broader panel of 24 isolates (Fig. S6). Chromosomal amplifications were surprisingly stable, however, and lingered in the population out to day 10 (~200 total generations). The slow depletion of the PMB-resistant subpopulation is consistent with a modest fitness cost for amplifying 145-kb (C1) and 264-kb (C2) genome segments flanking the *arn* operon. Indeed, monoculture growth rates were only modestly compromised in LB media in comparison to the Parent strain (Fig. S7A and S7B). Co-culture competition outgrowth assays did confirm a slight fitness cost for amplification (Fig. S7C and S7D), particularly apparent for the C2 isolate whose amplified chromosomal segment is longer and at a higher copy number in comparison to C1. At sub-MIC PMB concentrations, however, both C1 and C2 were stably maintained in the culture population.

### IS*1*-18 is necessary for chromosomal amplification and is the dominant route of PMB resistance in the absence of PEtN modification

**Fig 4:**
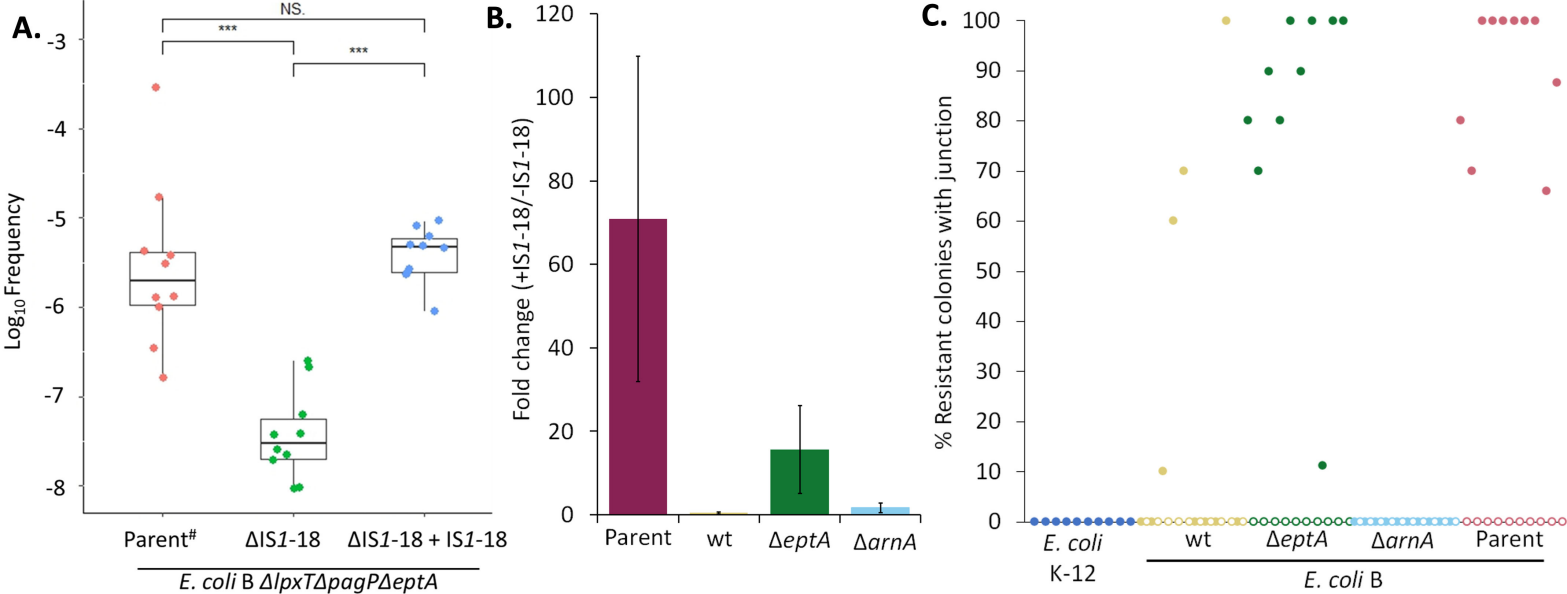
IS*1*-18-mediated chromosomal amplification of segments containing the *arn* operon increases the frequency of PMB resistance. (A) Frequency of resistance was determined for Parent strain, Parent strain lacking IS*1*-18 (ΔIS*1*-18), and with IS*1*-18 reinserted. The FOR of 10 separate cultures per strain was obtained by plating dilutions of exponential growths (OD_600_ of 0.2 to 0.7) on LBA containing 1 µg/mL PMB and then measuring the ratio of resistant CFUs to total plated cells. Significant values are based on Student’s *t*-test (***P < 0.001; NS, not significant). # indicates re-plotting of data shown in Fig. 1B. (B) Fold change in median FOR when deleting IS*1*-18 was quantified for the Parent, *E. coli* BL21(DE3) wt, Δ*eptA*, and Δ*arnA* strains. Error bars are derived from propagating median absolute deviation of values in the interquartile range. (C) For each *E. coli* B strain [with (filled circles) or without (open circles) IS*1*-18], PMB-resistant colonies were obtained from ~10 separate cultures. Within each PMB-resistant plating, up to 10 random colonies were sampled by multiplex junction PCR to estimate the percentage of PMB-resistant colonies arising by gene amplification (up to 100 resistant colonies per strain). *E. coli* K-12, which naturally lacks IS*1*-18, is shown for comparison.

The majority of PMB-resistant isolates utilized IS*1*-18 to amplify the *arn* operon, which is the closest downstream element (located ~90 kb from the end of the *arn* operon; see Fig. 3D). To directly investigate the role of IS*1*-18, we deleted the entire IS*1*-18 element and remeasured the FOR (Fig. 4). The FOR to PMB dropped nearly 70-fold, which could be reversed by reintroducing a copy of IS*1*-18 back into the same position on the chromosome. While IS*1*-18 significantly altered the overall FOR in the Parent strain, the effect of IS*1*-18-mediated amplification on the wild-type strain was less apparent (Fig. S8). The contribution of IS*1*-18 to the total PMB-resistant subpopulation, however, could be readily observed when *eptA* was deleted (~15-fold difference in FOR for IS*1*-18^+^ versus IS*1*-18^−^). In all backgrounds, the observed FOR was IS*1*-18 independent when the *arn* operon was deleted (Fig. S8; Fig. 4B). Since FOR records all types of PMB resistance mechanisms, we utilized the abovementioned multiplex junction PCR to determine the fraction of resistant colonies arising through IS*1*-18-mediated amplification events (Fig. 4C). In strains lacking either IS*1*-18 or an intact *arn* operon, amplification was not detected in any of the resistant colonies. In the wild-type background, resistance could be attributed to chromosomal amplification in approximately 30% of cases (*n* = 74 total colonies, up to 10 colonies checked from each of 10 replicates). There was an apparent bi-modal distribution, whereby certain replicate cultures were highly enriched in IS*1*-18-mediated amplifications. This suggests that *arn* operon amplification is not the sole mechanism of resistance and is consistent with a minor contribution to the overall FOR (Fig. 4B). However, in Δ*eptA* strain backgrounds where PEtN-mediated resistance mechanisms are prevented and there is no competition for LPS substrate, chromosomal amplification of the *arn* operon could be confidently assigned in ~90% of the PMB-resistant subpopulation.

### IS*1-*mediated PMB resistance is conserved among *E. coli* B lineages

IS*1* elements flank the *arn* operon in *E. coli* BL21(DE3), whereas they are absent from *E. coli* K-12 (Fig. S9A). To more generalize our observations, we identified other *E. coli* B strains with a similar distribution of IS*1* elements. *E. coli* ATCC 11303 has 28 IS*1* elements, 27 of which are shared with *E. coli* BL21(DE3) and most importantly include IS*1*-16, IS*1*-17, and IS*1*-18 (58). The PMB FOR was determined in otherwise isogenic strains of *E. coli* B ATCC 11303 (Fig. S9B). As in *E. coli* BL21(DE3) (Fig. 4; Fig. S8), deletion of IS*1*-18 specifically reduced the FOR ~14-fold in Δ*eptA* backgrounds. Collectively, the data support a model whereby (i) IS*1*-18 is required for efficient chromosomal amplification of DNA segments encoding the *arn* operon, (ii) phenotypic PMB resistance through amplification depends on a functional *arn* system, and (iii) IS*1*-18 amplification is the primary route of resistance without PEtN but is one of multiple, relevant mechanisms in *eptA*^+^ backgrounds.

### Chromosomal amplification results in global transcriptomic changes

**Fig 5:**
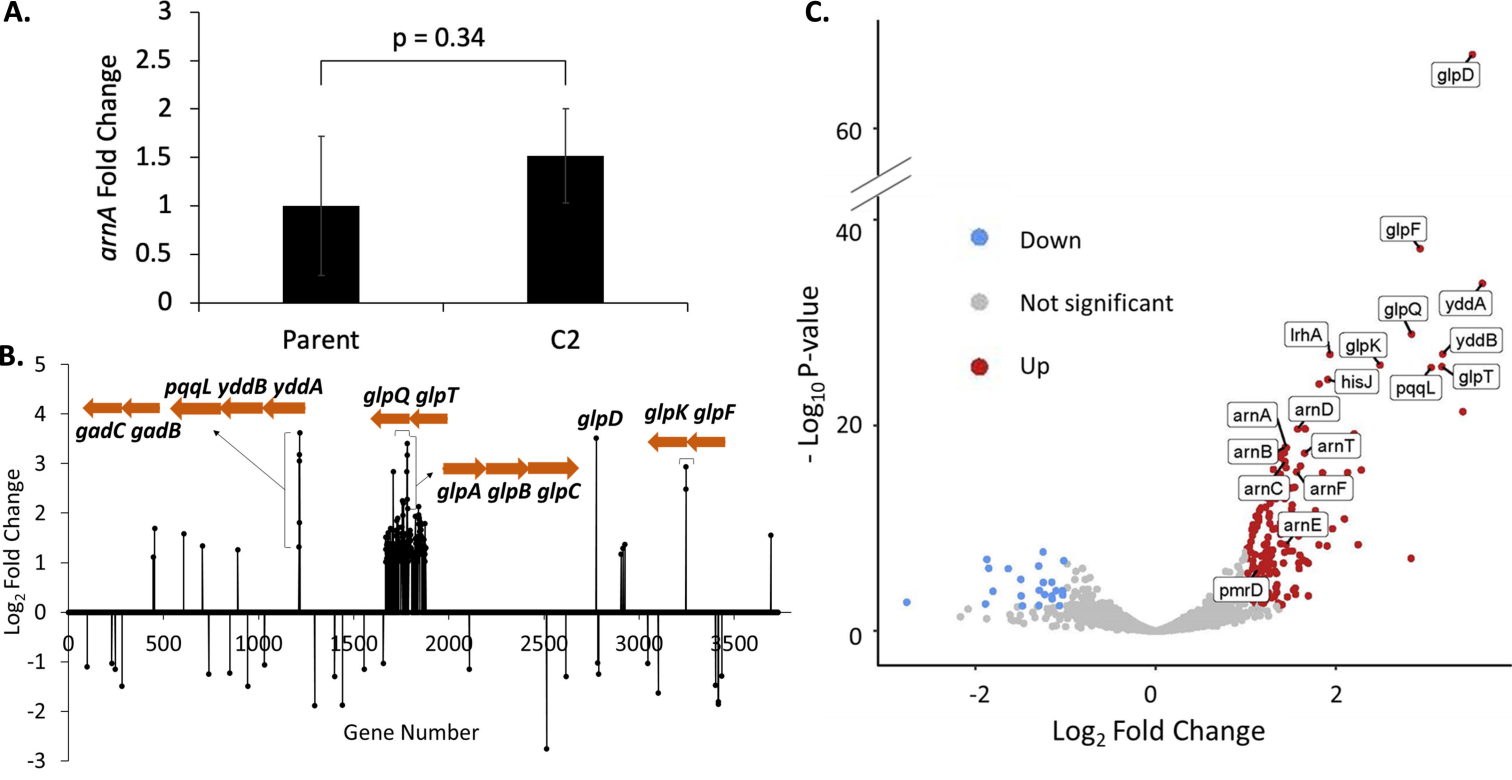
Chromosomal amplification results in global transcriptome changes. (A) RT-qPCR data for *arnA* expression in the Parent and C2 isolate. Expression is normalized to the *arcA* reference gene. Data represent *arnA* fold change values with respect to Parent for three independent experiments, performed in triplicate (*n* = 3 biological replicates for each strain). (B) Significant[false discovery rate (FDR) < 0.05] gene expression changes in C2 (log_2_ fold change ≥ 1 and ≤ −1 shown as is; remaining values collapsed to 0) compared with Parent are plotted against gene position along the chromosome. The amplified region appears as a cluster of upregulated genes containing the *arn* operon. Genes of interest *glpQT* and *glpABC* are present in the amplified region. Global transcriptomic changes include up- and downregulation of several operons, including phospholipid recycling gene clusters (*glpD*, *glpKF*) and a putative multidrug efflux pump (*yddAB-pqqL*). (C) Volcano plot from differential gene expression analysis showing significantly up- and downregulated genes. As expected, the *arn* operon is upregulated along with other genes residing outside the amplified region, including genes of interest *glpD*, *glpKF*, *yddAB*, and *pqqL* (*n* = 3 biological replicates for each strain).

While the *arn* operon copy number increased only by up to two- to threefold in C1 and C2, the PMB MIC increased ~20-fold. Considering the size of the amplifications (145 and 264 kb of DNA, respectively), it was not clear whether the seemingly disproportionate increase in the MIC was due to changes in the expression levels of the *arn* operon alone or in combination with other regulatory factors. There is little information regarding how genome amplification events may modulate genome-wide expression profiles. Upregulation of genes within the amplified segment might be expected, but potential effects on genes residing outside the duplication are difficult to predict. To provide insight, we isolated the total RNA from the representative mutant C2 and first performed reverse transcription quantitative real-time PCR (RT-qPCR) to quantify the *arnA* mRNA levels (Fig. 5A). Transcript levels of *arnA* were modestly higher than those in the Parent strain and in line with the respective gene copy number, ruling out any potential disconnect between gene copy and expression. We next performed RNA-seq to understand how amplification may impact global expression. When genes with significant fold changes (|log_2FC_| ≥1 and FDR < 0.05) are plotted against the gene position on the chromosome, a distinct profile for C2 in comparison to the Parent strain becomes apparent (Fig. 5B). There is a clear stretch of upregulated transcripts corresponding to the amplified segment, with sharply defined boundaries positioned at IS*1*-16/IS*1*-18. While the majority of genes within this segment were upregulated, the number of up- and downregulated genes was more balanced outside the amplicon (Fig. 5B). The most prominent change accompanying *arn* operon amplification involves the glycerol 3-phosphate (*glp*) regulon (Fig. 5C), a multi-operon pathway involved in glycerophospholipid catabolism (59). The *glp* regulon contains four separate transcripts, all of which were upregulated (divergently transcribed *glpTQ* and *glpABC* within the amplified segment, along with *glpD* and *glpFK* outside of the amplified segment) (Fig. 5B). Regulation of the *glp* pathway genes is complex, involving GlpR (note that its expression is unchanged), Crp, ArcA, Fnr, and *fnrs* sRNA [from Ecocyc database (60)]. The *yddAB-pqqL* locus was also upregulated in C2. The function of this operon is poorly understood, though it has been suggested to encode a multidrug efflux pump (61) and/or participate in iron acquisition (62). Regardless, the RNA-seq analysis provides a clear example of how IS*1*-mediated amplification induces genome-wide transcriptome changes to generate phenotypic strain diversity that includes PMB HR.

## DISCUSSION

While characterizing LPS extracted from strains of *E. coli* B (47, 48), we noticed high levels of intrinsic Ara4N modification when compared with *E. coli* K-12. The PMB MIC was also higher, albeit more variable, as would be expected with an outer membrane containing highly modified LPS. Intriguingly, the FOR at four to eight times their respective MICs was also nearly 100-fold higher in *E. coli* B (Fig. 1). While non-synonymous codon changes between the two-component regulator PmrAB systems may possibly explain the difference in the intrinsic resistance to PMB (40, 63), the *E. coli* B-specific mechanisms generating the higher FOR remain unknown. The FOR in *E. coli* B was minimally affected by deletion of *eptA* alone or in conjunction with *lpxT* and *pagP*. By contrast, the more than 10-fold drop in the FOR after deletion of *arnA* implicated the *arn* operon in creating the PMB-resistant subpopulation. Here, we have identified an IS*1*-mediated mechanism that increases Ara4N levels on LPS but is distinct from previously well-characterized PMB mutations. Chromosomal *arn* operon amplification was observed in all *E. coli* B strains tested, provided that the *arn* operon was intact and the flanking IS*1*-18 element was present (Fig. 1, 3 and 4; Fig. S9). LPS analysis by ^32^P-lipid A radiolabeling and ESI-MS revealed a bulk increase in LPS Ara4N content (Fig. 2; Data File S1). Amplification became the dominant mechanism in strains lacking PEtN. This likely reflects the expanded selection window, whereby a larger fraction of LPS substrate is available for Ara4N modification in comparison to *eptA*^+^ strains. Amplifications were relatively stable considering the large size (C1 at 145 kb and C2 at 264 kb), with PMB resistance at least partially retained for ~200 generations (Fig. 3E; Fig. S6). Duplications are not fixed and eventually do convert the resistant phenotype to a sensitive one—a hallmark of HR (32). However, the IS*1*-mediated amplified subpopulation could provide a reservoir for fixing mutations to accrue in two-component systems governing the addition of PEtN and Ara4N on LPS. This type of adaptive evolution is an integral component of gene amplification (64) and is frequently observed during serial passaging in the presence of antibiotics (65). Alternatively, resolution of the duplication once PMB selection passes to restore the wild type would avoid the fitness cost and attenuated virulence often associated with canonical PMB resistance mechanisms (66–74).

Chromosomal tandem duplications are among the most common class of mutations, and yet, their fleeting nature makes identification challenging (56). Tandem amplifications are thought to arise from unequal DNA exchange between sister chromosomes (Fig. 3B), resulting in a subpopulation that contains an additional copy of genes within that segment (57). While gene amplification leading to phenotypic antibiotic resistance has been recognized for many decades now (75–81), recent studies suggest gene amplification may be the dominant HR mechanism and account for up to 10% of all treatment failures (82). Among 41 isolates representing four opportunistic Gram-negative pathogens (*A. baumannii*, *E. coli*, *Klebsiella pneumoniae*, and *Salmonella enterica*), HR subpopulations were identified in a quarter of all strain-antibiotic combinations using a panel of 28 different antibiotics with diverse mechanisms of action (83). Within the phenotypically HR subset, over half could be conclusively linked to drug resistance gene amplification. Colistin and PMB were tested but HR was not detected in this study (83). However, PMB/colistin HR has been reported in certain strains of *A. baumannii*, *E. coli*, and *K. pneumoniae* (84–88) and an earlier study had identified *arn* operon amplifications in *S. enterica* by serially passaging at sub-MIC concentrations of colistin (89). Unlike in this study (Fig. 3D), the amplified segments from independent *S. enterica* isolates did not share a common junction and join points did not have any detectable DNA homology. Tandem amplification of chromosomal DNA segments is stimulated when flanked by repeats of homologous DNA, as with rRNA cistrons (90, 91) and IS elements (54, 55). Furthermore, it has been proposed that IS-encoded transposases themselves may stimulate amplification in addition to simply providing homologous DNA for recombination (54). HR driven by IS elements would therefore be expected to be highly strain specific, considering IS elements are mobile. In the case of *E. coli* B, the absence/presence of just a single IS*1*-18 can shift the apparent PMB FOR 10–100-fold.

In lineages with high IS-content such as *E. coli* B (Fig. S9A), the majority of the genome is potentially subject to amplification. Segments can be bounded by neighboring or non-contiguous IS elements located hundreds of kb apart (Fig. 3D), increasing both the phenotypic diversity and the overall size of the amplified subpopulation. While we did not pursue the causes of IS*1*-18-independent PMB resistance, IS*1*-mediated amplification of other chromosomal regions involved in PEtN addition or *arn* operon regulation may impart phenotypic PMB HR. RNA-seq analysis of the C2 isolate demonstrated transcriptional changes need not be confined to genes residing within the amplified segment (Fig. 5). The most robust change in expression involved the glycerophospholipid degradation (*glp*) pathway, which has genes located inside and outside the duplicated segment (Fig. 5B and C). Whether these changes directly contribute to PMB resistance is unclear, as the addition of the *arn* operon alone on a low-copy plasmid in the Parent strain phenocopies PMB resistance in the amplified mutants (Table S1). This does not rule out more subtle contributions to drug resistance, as overexpression of either the aerobic (*glpD*) or anaerobic (*glpABC*) *sn*-glycerol 3-phosphate dehydrogenase has been reported to induce multidrug tolerance in *E. coli* (92).

Resistance to polymyxins is a complex and growing problem (4, 5, 7). Alterations in 14 different genes encompassing LPS biosynthesis and modification, protein regulators/adaptors, two-component signaling systems, global transcriptional regulators, outer membrane porins, and efflux pumps have been detected in naturally occurring polymyxin-resistant *Enterobacterales* of animal or human origin (reviewed in reference 93). This study demonstrates how IS*1*-driven chromosomal *arn* operon amplification increases Ara4N modification of lipid A and contributes to the frequency of polymyxin HR in *E. coli* B *in vitro*. The IS content and site of insertion, which can act from hundreds of kilobases away, are rarely considered as a potential contributing factor in clinical isolates. To what extent IS-mediated genome amplification of the *arn* operon either directly contributes to clinical polymyxin resistance or generates a transiently resistant subpopulation for stably inherited resistant alleles to arise is currently unknown. In strains with fortuitously positioned IS*1* elements flanking the *arn* operon, however, gene amplification can clearly confound polymyxin susceptibility testing.

## MATERIALS AND METHODS

### Bacterial strains and growth conditions

*E. coli* K-12, BL21(DE3), and ATCC 11303 strains used in this study were grown in lysogeny broth (LB, 10 g tryptone, 10 g NaCl, 5 g yeast extract, pH 7.0) only or in LB supplemented with appropriate antibiotics. Standard culture conditions were defined as 2-mL cultures in 14-mL Falcon snap cap culture tubes, grown at 37°C with aeration (250 rpm). Where noted, cultures were grown in 96-well plates and 24-well plates, with or without shaking. Plasmids and strains used in this study are listed in Table S2.

### Strain and plasmid construction

Gene or IS*1*-18 deletions along with the IS*1*-18 knockin back complement were constructed using the Red recombinase system (94). Gene-specific P1 and P2 primers, along with the antibiotic resistance gene marker template used for selection, are listed in Table S3. In the case of ΔIS*1*-18, an apramycin (*apr*) marker was amplified by PCR (primers MM2891-2892) and flanked by DNA cassettes located up- (primers MM2896-2897) and downstream of IS*1*-18 (primers MM2898-2899). PCR products were assembled in the plasmid pKFC, which was then used as template for PCR amplification (primers MM2900-2901) of the integration cassette. For IS*1*-18 knockin to make the back-complemented strain, splice overlap PCR was used to assemble the upstream DNA homology arm (primers TM3013-3018), IS*1-*18 (TM2983-2984), a spectinomycin resistance selection marker (TM3055-3056), and a downstream homology corresponding to the *apr* marker (TM3014-2019). All constructs were checked by PCR using primers annealing outside the integration locus for the correct size before verification by Sanger sequencing. The low-copy *arn* operon plasmid, pCL25*-arn* op, was constructed by amplifying the pCL25 plasmid backbone (primers TXM2929-2930) and using the In Fusion Snap Assembly Master Mix (Takara Bio) to introduce the entire *arn-pmrD* operon that was amplified from *E. coli* BL21(DE3) (primers MM2864 and MM2908).

### Population analysis profile

Single colonies of wild-type *E. coli* BL21(DE3) (TXM319) were inoculated in 2 mL of LB in 15-mL culture tubes. From the overnight culture, 3 mL of LB inoculated with 100 µL of a 10^6^-fold dilution was incubated at 37°C with shaking till cells reached early exponential growth phase (OD_600_ of 0.15–0.3). Cells were harvested by centrifugation (2 min at 5,000 × *g*) and resuspended in an equal volume of LB with PMB (0.5 µg/mL). Aliquots containing 10^7^, 10^6^, and 10^5^ cells were plated on LBA (LB plus 1.5% agar, pH 7) with varying concentrations of PMB (0, 0.5, 1, 2, 4, and 6 µg/mL). Colony counts were obtained after an overnight incubation at 37°C and used to calculate the FOR at the corresponding concentration of PMB.

### MIC, growth curve measurements, and competition assay

For MIC measurements, a low CFU input was used to minimize the effect of resistant subpopulations. Single colonies of strains isolated on LBA, LBA supplemented with 0.5 µg/mL PMB (for PMB-resistant isolates), or 50 µg/mL spectinomycin (strains with pCL25-*arn* op) were cultured overnight under standard culture conditions at 37°C in 2 mL of LB or LB supplemented with appropriate antibiotics. An inoculum of ~100 CFU was added to each well in a 96-well microplate with increasing PMB (twofold increments) concentrations in LB and incubated overnight at 37°C without shaking. The minimum concentration of PMB with no visible growth was scored as the MIC after 18–20 hours of incubation. For growth curve measurements, overnight cultures of Parent and mutant strains were diluted 1:1,000 in 2 mL of fresh LB in 15-mL culture tubes and incubated at 37°C with aeration by shaking (250 rpm). Growth was measured at OD_600_ with readings taken every 30 min. For competition assay, overnight cultures of Parent and mutant strains were diluted 1:1,000 in 2 mL fresh LB and incubated at 37°C with shaking till the early exponential phase. Equal CFUs of Parent and mutant were added to LB and LB with 0.05 µg/mL PMB and incubated at 37°C with shaking and passaged over 5 days. Appropriate monocultures were set up as controls. Aliquots of 5 µL were collected each day and mixed with 45 µL of lysis buffer [10 mM Tris-HCl, 0.1 mM EDTA, 0.1% Triton X-100 (pH 8.0)]. After heating at 95°C for 10 min, 1 µL of this dilution was used as template for Junction multiplex PCR as described. The band intensities of control (*pykA*) and amplification (P17-18 or P16-18) were measured using ImageJ (95).

### Determination of the frequency of resistance

For each strain, glycerol stocks were struck out on to LBA with required antibiotics for marker selection. Ten single colonies were randomly selected to inoculate separate LB cultures and grown overnight. The next morning, 100 µL of a 10^6^ dilution was used to inoculate a fresh 3-mL LB culture and incubated at 37°C until an OD_600_ between 0.2 and 0.7. Cells were pelleted (2 min at 5,000 *× g*) and then resuspended in an equal volume of LB containing either 0.5 or 1 µg/mL PMB (the latter was used for strains with MIC values consistently above 1 µg/mL). Each resuspension was serially 10-fold diluted, and 100 µL of each was plated on separate LB agar plates containing PMB at four- to eightfold higher than the MIC. The fold change is approximated as replicate MIC measurements for PMB were highly variable (See Table S1). The FOR was calculated based on the ratio of CFU on a PMB-containing plate over the total CFU count (based on the LBA-only plate).

### Single-step derivation of independent PMB-resistant isolates

To derive PMB-resistant isolates, single colonies of the Parent strain AR1973 [*E. coli* BL21(DE3) *eptA::catR lpxT::kanR pagP::hygR*)] were used to start independent overnight cultures. Cultures were diluted into LB supplemented with increasing concentrations of PMB (final concentration range in culture was 0.03 μg/mL to 0.5 μg/mL) in a 24-well microplate, with a final culture volume of 3 mL at 10^6^ CFU/mL to enrich the PMB HR subpopulation. The plate was incubated at 37°C with shaking (250 rpm) for 24 hours. Growth was observed for all cultures at the highest concentration (0.5 µg/mL). Cultures were colony purified on LBA with PMB (0.5 µg/mL) and used to make glycerol stocks. In all cases, phenotypic PMB resistance was stable, provided that cultures were maintained on PMB, as determined by MIC measurements (2–4 µg/mL PMB) and FOR (1 µg/mL PMB) performed as described above.

### LPS purification

Bacteria were harvested from stationary phase cultures grown at 37°C in LB supplemented with necessary antibiotics for genetic marker selection. PMB was not included to avoid any possible further selection during culture. LPS was isolated as previously described (47, 48). Dried biomass from 4 L of culture was gathered by stirring with ethanol overnight followed by two 12-hour rounds with acetone at 4°C. From dried biomass, LPS was extracted via the phenol-chloroform-petroleum ether method (96). From the PCP-extracted LPS, contaminating phospholipids were removed by a modified chloroform-methanol wash (97). Briefly, pellets were resuspended in chloroform/methanol/3 M sodium acetate (pH 7.0) (85:15:1, vol/vol/vol) and LPS was precipitated via the addition of 2–3 volumes of methanol and collected by centrifugation (10 min at 3,000 × *g*). The wash was repeated twice more before lipoprotein contamination was removed following the phenol/sodium deoxycholate extraction as described by Hirschfeld et al. (98), but using a Tris-buffered, pH neutral TEA-DOC solution [0.5% sodium deoxycholate (wt/vol), 0.2% triethylamine (vol/vol), in 20 mM Tris-HCl, (pH 7.0)]. Nucleic acid contamination was removed by resuspending LPS pellets in 3 mL of water and diluted with 30 mL of Tris-saline buffer (50 mM Tris-HCl, 100 mM NaCl, 1 mM MgCl_2_, pH 7.0) before centrifugation at 100,000 × *g* for 4 hours at 4°C. Supernatant was decanted and suspended in water before transfer to 500–1,000-Da MWCO dialysis tubing. LPS was dialyzed against four 5-L portions of Milli-Q water at 4°C for 48 hours. Finally, LPS was lyophilized and stored at –20°C for ESI-MS analysis.

### Mild hydrolysis of LPS

Lipid A and core oligosaccharides were prepared from LPS samples by mild hydrolysis as described (99), with slight modifications. Briefly, LPS samples (5–7 mg) were dissolved in water at a final concentration of 6.3 mg/mL, and a solution of 10% SDS (volume equivalent to 12.5% of the water volume) along with an equivalent volume of acetate buffer (1 M NaOAc, pH 4.4) was added. The mixture was heated for 30 min at 100°C under slight stirring and afterward freeze dried. SDS was removed by four washes with 4 mL 2 M HCl/EtOH (1:99, vol/vol) (centrifugation: 6,000 × *g* for 20 min at 20°C). The dried pellet was resuspended in 1.5 mL water. Afterward, 1.5-mL aliquots of a CHCl_3_/CH_3_OH mixture (4:1 vol/vol) were added and the suspension was mixed vigorously and centrifuged (6,000 × *g*) for 10 min at 4°C. The organic phase was collected, and the water phase (including the interphase) was extracted again three times with 1.5 mL CHCl_3_. All organic phases were combined and dried under a stream of nitrogen. The core oligosaccharide-containing water phase was lyophilized and dialyzed against water for 3 days at 4°C with six water exchanges in total (MWCO: 500–1,000 Da; Biotech-Grade CE Dialysis Tubing, Spectrum Laboratories Inc., Rancho Dominguez, CA, USA). Samples were then analyzed directly by ESI-MS without further purification.

### Electrospray ionization mass spectrometry

Mass spectrometric analyses of LPS and core oligosaccharide samples were performed on a QExactive Plus mass spectrometer (Thermo Scientific, Bremen, Germany) using a Triversa Nanomate platform (Advion, Ithaca, NY, USA) as the nano-ESI source. LPS samples were initially dissolved in water in a concentration of 1 µg/µL, and 5 µL of these solutions was mixed with 95 µL of MS solvent [water, 2-propanol, 7 M trimethylamine, acetic acid (50:50:0.06:0.02, vol/vol/vol/vol)]. Lipid A [1 µg/µL solutions in CHCl_3_, CH_3_OH, water (60:30:4.5, vol/vol/vol); 5 µL with 95 µL MS solvent] and core oligosaccharides (10 µg/mL solutions in water; 2 µL with 98 µL MS solvent) were likewise prepared. All samples were centrifuged briefly prior application to the MS. Mass spectra were recorded for 0.50 min in the negative-ion mode in an *m*/*z* range of 400 to 2,500 applying a spray voltage set to –1.1 kV. Mass spectra were charge deconvoluted [Xtract module of the Xcalibur 3.1 software (Thermo Fisher Scientific, Bremen, Germany)], and the given mass values refer to the monoisotopic masses of the neutral molecules, if not stated otherwise. For the calculation of relative ratios of non-modified and Ara4N-modified LPS species, mass peak intensities of the deconvoluted spectra depicted in Fig. 2A; Fig. S2 were taken. For all molecules, the intensity of the first isotopic peak was considered. All LPS-related peaks for which the relative intensity of the corresponding peak was above 10% in comparison to the most abundant LPS molecule in at least one of the spectra were included in the calculation (Data File S1).

### Labeling of lipid A with ^32^P and analyses via thin layer chromatography

Radiolabeled lipid A was extracted from strains according to published procedures (100). Overnight cultures for each strain were prepared in LB with selection marker antibiotics as required. Isolates were grown without PMB challenge and used to inoculate 7 mL of LB by 1:100 (vol/vol) dilution supplemented with inorganic ^32^P at 2.5 µCi/mL. Cells were grown for ~4 hours at 37°C with shaking at 250 rpm. Cells were harvested by centrifugation at 2,000 × *g* for 10 min and washed with water before resuspension in 1 mL of a single-phase Bligh-Dyer mixture [chloroform/methanol/water (1:2:0.8, vol/vol/vol)]. Suspended cells were allowed to incubate at room temperature with inversion to mix for 15 min before pelleting (6,000 × *g* for 2 min) and resuspension with a second aliquot of single-phase Bligh-Dyer mixture. The incubation period was repeated, LPS pelleted, and resuspended in 450 µL of 300 mM sodium acetate (pH 4.5)/1% SDS buffer by pipetting. Lipid A hydrolysis was achieved by incubating samples at 95°C for 45 min. After hydrolysis, samples were allowed to cool before conversion to a two-phase Bligh-Dyer mixture via the addition of 1 mL of chloroform:methanol (1:1, vol/vol) for a final chloroform:methanol:water (2:2:1.8, vol/vol) mixture. The two-phase Bligh-Dyer was vortexed, and phases were separated via centrifugation (3,000 × *g* for 10 seconds). The lower phase containing ^32^P-labeled lipid A was extracted into a clean Eppendorf tube and allowed to dry overnight.

To visualize ^32^P-labeled lipid A, samples were dissolved in 50 µL of chloroform:methanol (4:1 vol/vol) and 5 µL spotted on a silica gel 60 TLC plate (Merck Millpore Catalogue# 1.05721.0001). Plates were developed using a pyridine/chloroform/formic acid/water (50:50:16:5, vol/vol/vol/vol) mobile phase and allowed to air dry overnight. A Phosphorimager screen [BAS-IP MS (Multipurpose Standard)] was exposed to the plastic-wrapped TLC plate for 24 hours before scanning using a Typhoon 9400.

### Whole genome sequencing

From 1 mL of overnight cultures in LB, genomic DNA was extracted using guanidinium thiocyanate as described (101). Genomic DNA was submitted to SeqCenter (Pittsburgh, PA) for Illumina next-generation sequencing (1.33 million reads per sample using paired end 150 bp long short end reads). Reads were mapped to the reference genome of *E. coli* BL21(DE3) (NCBI Reference Sequence: NC_012971.2) using Geneious Prime software (https://www.geneious.com, version 2023.0.1).

### Resolution of amplified chromosomal segments

Single colonies of Parent and PMB-resistant isolates were struck on LBA or LBA supplemented with 0.5 µg/mL PMB, respectively, and were inoculated in 200 µL of LB media either without (for Parent) or with 1 µg/mL PMB in a 96-well microplate. Cultures were incubated overnight at 37°C, without shaking. For the first passage, a 10^5^-fold dilution of overnight culture was made into 2 mL of LB or LB supplemented with 0.5 µg/mL PMB in 15-mL culture tubes and incubated overnight at 37°C, with shaking (250 rpm). After 2 hours of incubation, each sample was plated on LBA and LBA containing 0.5 µg/mL PMB to verify the corresponding homogeneous susceptible (Parent) or resistant PMB (C1 and C2) phenotypes. Passage 2 onwards, a 10^7^-fold dilution of stationary phase cultures was performed into final 2-mL culture volumes (with or without 1 µg/mL PMB). The proportion of PMB-resistant colonies was calculated as the ratio of colony counts from LBA with 1 µg/mL PMB plates to those from LBA-only plates for each sample per passage per day. Approximately 20 generations occurred in each daily passage.

### Junction multiplex PCR

Tandem chromosomal duplication was checked by multiplex PCR using whole cell colony PCR with Thermo Scientific Phire Plant Direct PCR Master Mix and primers annealing next to IS*1*-16, -17, and -18 (P16, P17, and P18, Table S3). Primers (pkyAfor/pykArev) annealing to a control locus located outside of the amplified regions were used as internal controls. In ΔIS*1-*18 strains, primer P18 was replaced with primer P18′. Template DNA was prepared via genomic DNA extraction as described above or directly from isolated single colonies resuspended in 10 µL of lysis buffer [10 mM Tris-HCl, 0.1 mM EDTA, 0.1% Triton X-100 (pH 8.0)]. Samples were incubated for 5 min at 95°C and then centrifuged for 10 min at 13,000 × *g*. Aliquots of 0.5 µL of resultant supernatant were used as template. PCR conditions followed recommendations provided by the manufacturer, with an annealing temperature of 60°C and an extension time of 40 seconds at 72°C.

### Total RNA isolation

Single colonies of Parent and of the PMB-resistant isolates C1 or C2 were obtained on LBA and LBA supplemented with 0.5 µg/mL PMB. Single colonies were used to start overnight cultures in 200 µL of LB in microwell plates at 37°C, without shaking. The following day, a 1:1,000 dilution of overnight culture in 3 mL of LB was incubated with shaking at 37°C. Cell pellets were harvested from 1 mL of culture at OD_600_ of 1.0. The pellet was resuspended in 900 µL of TRI Reagent (Sigma Aldrich) and stored at –80°C until RNA extraction. RNA extraction was performed using a PureLink RNA Mini Kit (Invitrogen) with the following modifications. Equal volume of 0.1 mm zirconium beads was added to the thawed cells in TRI Reagent, and cells were lysed by bead-beating (Biospec minibead beater) at 3,800 rpm for three 30-second cycles with a 3-min rest on ice in between. Beads and unbroken cells were removed by centrifugation (3,000 × *g* for 2 min), and 500 µL of each supernatant was mixed with chloroform (100 µL) to induce phase separation before centrifugation (12,000 × *g* for 15 min at 4°C). A 400 µL aliquot of the upper aqueous phase was mixed with an equal volume of 70% ethanol, and the entire volume was transferred to a silica spin column for RNA isolation according to the manufacturer’s instructions. Total RNA was eluted with 100 µL of RNase-free water and quantified by absorbance at 260 nm.

### RNA-seq analysis

RNA samples isolated as described above were submitted to SeqCenter (Pittsburgh, PA) for RNA-seq (12 million reads per sample using paired end 150-bp long short end reads) according to submission guidelines. Resulting transcript counts of 10 or higher were mapped to the *E. coli* BL21(DE3) reference sequence (NCBI Reference Sequence: NC_012971.2). Differential gene expression analysis was performed using Bioconductor’s edgeR package in R (102, 103).

### RT-qPCR

Total RNA isolated as above was further purified by treatment with DNase treatment (Thermo Fisher Scientific TURBO DNA-free Kit). RT-qPCR was performed using a Luna Universal One-Step RT-qPCR Kit (NEB) on DNase-treated RNA samples (0.8 µg per sample), with gene-qPCR-specific primer pairs (see Table S3), using an Applied Biosystems 7300 real-time PCR machine. All data were measured in triplicate and normalized to the validated housekeeping internal control gene *arcA* (104), and relative expression levels were calculated using the 2^−ΔΔCT^ method (105).

### SDS-PAGE separation and silver staining of LPS

Whole cell lysates were prepared according to Hitchcock and Brown (106), with the following modifications (107, 108). Bacteria were grown overnight in 2 mL of LB with required antibiotics, and C1 and C2 isolates were grown in the absence of PMB. From the overnight growth, 100 µL was pelleted (5,000 × *g* for 2 min). The pellet was solubilized in 50 µL of lysing/loading buffer (0.5 mM EDTA, 2% SDS, 2% 2-mercaptoethanol, 0.1% bromophenol blue, 10% glycerol, 50 mM Tris-HCl, pH 7.5). LPS powder purified as described above was resuspended in lysing/loading buffer to a concentration of 20 µg/mL. Lysates were incubated at 95°C for 10 min and cooled, and proteinase K (PK) was added to a final concentration of 1 µg/µL and incubated at 60°C for 60 min. Samples were resolved using 16.5% T, 6% C Tris-tricine SDS-PAGE gels according to Lesse et al. (109) and LPS visualized by silver staining (106).

## SUPPLEMENTAL MATERIAL

File S1 mbio.00634-24-s0001.xlsx LPS peak assignment, peak intensity, and relative quantitation of LPS Ara4N content.

Supplemental Tables and Figures mbio.00634-24-s0002.pdf Tables S1-S3 and Figures S1-S9.

ASM does not own the copyrights to Supplemental Material that may be linked to, or accessed through, an article. The authors have granted ASM a non-exclusive, world-wide license to publish the Supplemental Material files. Please contact the corresponding author directly for reuse.
