# Supplementary material for: IS1-mediated chromosomal amplification of the arn operon leads to polymyxin B resistance in Escherichia coli B strains
Source: mBio. 2024 Jun 21;15(7):e00634-24. doi: 10.1128/mbio.00634-24 (PMC11253626; doi:10.1128/mbio.00634-24)
Supplement: Supplemental Tables and Figures — Tables S1-S3 and Figures S1-S9. [file mbio.00634-24-s0002.pdf]

SUPPLEMENTARY MATERIALS

IS*1*-mediated chromosomal amplification of the *arn* operon leads to polymyxin B resistance in *Escherichia coli* B strains

Michael Maybin[a*], Aditi M. Ranade[b*], Ursula Schombel[c], Nicolas Gisch[c], Uwe Mamat[d], and Timothy C. Meredith[a,b]#

[a] Department of Biochemistry and Molecular Biology, The Pennsylvania State University, University Park, Pennsylvania, USA
[b] The Huck Institutes of the Life Sciences, The Pennsylvania State University, University Park, Pennsylvania, USA
[c] Division of Bioanalytical Chemistry, Priority Research Area Infections, Research Center Borstel, Leibniz Lung Center, 23845 Borstel, Germany
[d] Division of Cellular Microbiology, Priority Research Area Infections, Research Center Borstel, Leibniz Lung Center, Leibniz Research Alliance INFECTIONS, 23845 Borstel, Germany
*These authors contributed equally to this work and share first authorship.

Table S1: Polymyxin B (PMB) minimum inhibitory concentration (MIC) values[a]

Strain	Genotype	MIC (μg/mL)[b]			
		REP1	REP 2	REP 3	Mean
TXM338	Wildtype BW25113; K-12 lineage	0.06	0.06	0.06	0.06
TXM319	Wildtype BL21(DE3); B lineage	2	1	2	1.6
TXM322	BL21(DE3) *arnA*::*kanR*	0.06	0.06	0.06	0.06
GKM329	BL21(DE3) *eptA*::*catR*	0.06	0.06	0.06	0.06
AR1973 (Parent)	BL21(DE3) *eptA::catR lpxT::kanR pagP::hygR*	0.13	0.13	0.06	0.10
MM2028	BL21(DE3) *eptA::catR lpxT::FRT pagP::hygR arnA::kanR*	0.06	0.06	0.06	0.06
C1	BL21(DE3) *eptA::catR lpxT::kanR pagP::hygR*	2	2	2	2
C2	BL21(DE3 *eptA::catR lpxT::kanR pagP::hygR*	2	2	2	2
AR2206	BL21(DE3) IS*1*-18::*aprR*	2	4	4	3.3
AR2207	BL21(DE3) *arnA*::*kanR* IS*1*-18*::aprR*	0.06	0.06	0.06	0.06
AR2208	BL21(DE3) *eptA*::*catR* IS*1*-18*::aprR*	0.13	0.06	0.13	0.10
MM2165	BL21(DE3) *eptA::catR lpxT::kanR pagP::hygR* IS*1*-18::*aprR*	0.13	0.06	0.06	0.08
TXM2253	BL21(DE3) *eptA::catR lpxT::kanR pagP::hygR* IS*1*-18*::aprR* + IS*1*-18 *specR*	0.13	0.13	0.06	0.10
TXM2197	Wildtype *E. coli* (Miguela) Castellani and Chalmers ATCC11303	4	4	4	4
TXM2222	ATCC11303 *eptA::catR*	0.03	0.06	0.03	0.04
TXM2223	ATCC11303 *arnA::kanR*	0.03	0.03	0.06	0.04
MM2176	ATCC11303 IS*1-18::aprR*	4	4	4	4
TXM2233	ATCC11303 *eptA::catR* IS*1*-18*::aprR*	0.06	0.06	0.03	0.05
TXM2234	ATCC11303 *arnA::kanR* IS*1*-18*::aprR*	0.06	0.03	0.03	0.04
AR2318	Wildtype BL21 (DE3) + pCL25- *arn* op	4	8	4	5.3
AR2319	BL21(DE3) *arnA::kanR* + pCL25- *arn* op	8	8	8	8
AR2320	BL21(DE3) *eptA::catR* + pCL25- *arn* op	4	4	4	4
AR2321	BL21(DE3) *lpxT::kanR pagP::hygR* + pCL25- *arn* op	8	8	8	8
AR2322	BL21(DE3) *eptA::catR lpxT::kanR pagP::hygR* + pCL25- *arn* op	4	4	4	4
AR2323	BL21(DE3) *eptA::catR lpxT::FRT pagP::hygR arnA::kanR* + pCL25- *arn* op	4	4	4	4
AR2324	Wildtype *E. coli* (Miguela) Castellani and Chalmers ATCC11303 + pCL25- *arn* op	4	4	4	4
AR2325	ATCC11303 *eptA::catR* + pCL25- *arn* op	8	8	8	8
AR2326	ATCC11303 *arnA::kanR* + pCL25- *arn* op	8	8	8	8

[a] MIC values were measured with a low initial inoculum as described in Material and Methods. [b] Rep- biological replicate.

Table S2: Bacterial strains and plasmids

Bacterial Strain or plasmid	Relevant Genotype/Phenotype	Source or reference
***E. coli* strains**		
TXM338	Wildtype BW25113; K-12 lineage	Lab Stock
TXM319	Wildtype BL21 (DE3); B lineage	Lab Stock
TXM322	BL21(DE3) *arnA*::*kanR*; Kan^r	(1)
GKM329	BL21(DE3) *eptA*::*catR*; Cat^r	(1)
TXM331	BL21(DE3) *eptA*::*catR* *arnA*::*kanR*; Cat^r Kan^r	(1)
TXM333	BL21(DE3) *eptA*::*catR* *arnA*::*kanR* *lpcA*::*gentR*; Cat^r Kan^r Gent^r	(1)
AR2206	BL21(DE3) IS*1*-18::*aprR*; Apr^r	This study
AR2207	BL21(DE3) *arnA*::*kanR* IS*1*-18*::aprR*; Kan^r Apr^r	This study
AR2208	BL21(DE3) *eptA*::*catR* IS*1*-18*::aprR*; Cat^r Apr^r	This study
AR1972	BL21(DE3) *lpxT*::*kanR* *pagP*::*hygR*; Kan^r Hyg^r	This study
AR1973 (Parent)	BL21(DE3) *eptA*::*catR* *lpxT*::*kanR* *pagP*::*hygR*; Cat^r Kan^r Hyg^r	This study
MM2028	BL21(DE3) *eptA*::*catR* *lpxT*::FRT *pagP*::hygR *arn*A::*kanR*; Cat^r Kan^r Hyg^r	This study
MM2165	BL21(DE3) *eptA::catR lpxT::kanR pagP::hygR* IS*1*-18::*aprR*; Cat^r Kan^r Hyg^r Apr^r	This study
TXM2253	BL21(DE3) *eptA::catR lpxT::kanR pagP::hygR* IS*1*-18*::aprR* + IS*1*-18 *specR*; Cat^r Kan^r Hyg^r Apr^r Spec^r	This study
TXM2197	Wildtype *E. coli* (Migula) Castellani and Chalmers ATCC11303	ATCC
TXM2222	ATCC11303 *eptA*::*catR*; Cat^r	This study
TXM2223	ATCC11303 *arnA*::*kanR*; Kan^r	This study
MM2176	ATCC11303 IS*1*-18::*aprR*; Apr^r	This study
TXM2233	ATCC11303 *eptA*::*catR* IS*1*-18::*aprR*; Cat^r Apr^r	This study
TXM2234	ATCC11303 *arnA*::*kanR* IS*1*-18::*aprR*; Kan^r Apr^r	This study
AR2318	Wildtype BL21 (DE3) + pCL25- *arn* op; Spec^r	This study
AR2319	BL21(DE3) *arnA*::*kanR* + pCL25- *arn* op ; Kan^r Spec^r	This study
AR2320	BL21(DE3) *eptA*::*catR* + pCL25- *arn* op; Cat^r Spec^r	This study
AR2321	BL21(DE3) *lpxT::kanR pagP::hygR* + pCL25- *arn* op; Kan^r Hyg^r Spec^r	This study
AR2322	BL21(DE3) *eptA::catR lpxT::kanR pagP::hygR* + pCL25- *arn* op; Cat^r Kan^r Hyg^r Spec^r	This study
AR2323	BL21(DE3) *eptA::catR lpxT::FRT pagP::hygR arnA::kanR* + pCL25- *arn* op; Cat^r Kan^r Hyg^r Spec^r	This study
AR2324	Wildtype *E. coli* (Miguela) Castellani and Chalmers ATCC11303 + pCL25- *arn* op; Spec^r	This study
AR2325	ATCC11303 *eptA::catR* + pCL25- *arn* op; Cat^r Spec^r	This study
AR2326	ATCC11303 *arnA::kanR* + pCL25- *arn* op; Kan^r Spec^r	This study

**Plasmids**		
pKD46	λ-Red recombinase expression plasmid; $Carb^r$	(2)
pKD4	Kan^r template	(2)
pSET152	Apr^r template	Lab Stock
pKD3	Cat^r template	(2)
pUC19-oriT-hyg	Hyg^r template	Lab stock
pKFC	Vector for construction of LHA IS*1*-18::*aprR*	Lab stock
pCP20	FLP recombinase plasmid, $Carb^r$	(2)
pCL25	$Spec^r$ template	Lab Stock
pCL25-*arn* op	Vector for *arn* operon and *pmrD* expression (low copy); $Spec^r$	This study

Kan^r- kanamycin; Cat^r- chloramphenicol; $Spec^r$- spectinomycin; $Carb^r$- carbenicillin; Apr^r- apramycin; Hyg^r- hygromycin, $Gent^R$- gentamicin.

Table S3: Primers used in this study

Primer Name	Primer Sequence
**IS*1*-18 KO**	
MM2896-P1 Ins18 pKFC EcoRI	GACGGCCAGTGAATTCATGGAATAAAATCATGCTACC
MM2897-P2 Ins18 pKFC	TCGCTTTTCCCGCTCTCATCAAATTCCGTTACCG
MM2898-P3 Ins18 pKFC	AATCGCCAGGCGAAATATCAAAATGAAATTAATCAAC
MM2899-P4 Ins18 pKFC HindIII	TGATTACGCCAAGCTCGGATGGTCTTTACCCGGTTC
MM2891 - Ins18 aprR P1	GAGCGGGAAAAGCGAACCAACGGACTTATTTACCTGGTACGGTCACCTAGATCCTTTTGG
MM 2892 - Ins18 aprR P2	TTTCGCCTGGCGATTTTGAAGTCTTTTTTCAACGTTATCTTCCGTTCTCCGCTCATGAGC
MM2900-Ins18::aprR pKFC for	CAATACCAGCGCTGCTTACACAGA
MM2901-Ins18::aprR pKFC rev	CGATGTCTGTGGGTAAATGGACGC
MM2927-LHA Ins18 upcheck	GCTTACCCTTTCAAATCAATCAGTG
MM2928-LHA Ins18 downcheck	GTCCAGTTACCGTTCTACGGTAGC
**IS*1*-18 KI**	
TM3018- yfdF P1	GAAGTTAATGGATTAGTACAAGAGTTC
TM3013- yfdF P2	AACCAGTTGCAAATGCGAAGATAAAACCAGG
TM2983- Ins18 KI for	CATTTGCAACTGGTTCCGATCATC
TM2984-Ins18 KI rev	CCCGTTGAGCTCGTTTAC
TM3055- specIS18 for	AACGAGCTCAACGGGTTCCCCTGCTCGCGCAG
TM3056- specIS18 rev	GGTTTGCGAATCCGTGCTTGAACGAATTGTTAG
TM3014- apr P3	ACGGATTCGCAAACCGATATCACACAAACTCAGAC
TM3019- apr P4	CAGTCCAAGTGGCCCATCTTCGAG
***arnA* KO**	
GK425-ArnA::KanR-P1	GGTGACCTGCCTTACCACAAC
GK426-ArnA::KanR-P2	TCGTGATGTTTAGCCGCTTC
GK433-ArnA-check_for	CGAGCGTGAGTTTGGTGAATCC
GK434-ArnA-check_rev	CCGATCCCAGTTACCGCTAC
***eptA* KO**	
TM448-EptA::catR-P1	GTTGGCCGCTTTTTATATCTCTATCTGCCTGAATATTGCCTTGCGCCTACCTGTGACGGA
TM449-EptA::catR-P2	TGTTGCGTTTGCGCCTGTTTTTGCAGGCAGTTCTGGTCAACCCTTACGCCCCGCCCTGCC
GK435-EptA-check_for	AAACCCGTATCCCTTAGATGCACC
GK436-EptA_check_rev	CTCAAGGCTTTGTTCCGCCATC
***lpxT* KO**	
MAM2381- New P1 lpxT pKD4 KanR	AGGTTGCCTGCGTTTTTCAGTAAGATAATTAGAGAAAATATGGTAGGCTGGAGCTGCTTC

MAM2382- New P2 lpxT pKD4 KanR	GATGATGTTAATTACTGTGAGTTATTTGTTTTGGAAATGTTTTATGAATATCCTCCTTAG
MM2579- lpxT upcheck v2	CTGATTTCCAGCAGCGAAGCTGAC
MM2580- lpxT downcheck v2	GATAGATGAAAGCACGGTGCGCAT
***pagP* KO**	
Tm748- PagP hyg P1	GTTTTATGGTCACAAATGAACGTGAGTAAATATGTCGCTATCCTATGACCATGATTACGC
Tm749- PagP hyg P2	ACTAAAACTTCATTTGTCTCAAAACTGAAAGCGCATCCAGGCACGTTGTAAAACGACGGC
Tm750- PagP hygcheck for	GTAGCTTTGCTATGCTAGTAGTAG
Tm751- PagP hygcheck rev	GTGGTACGCTTTGTCCAGTGTAAC
**RT-qPCR**	
AR3070-arnA p1 RT qpcr	GAACGCATTGCCCAACTGTC
AR3071-arnA p2 RT qpcr	GTTTCACCGTTGACCAGCAC
AR3076-arcA p1 RT qpcr	ACCCCGCACATTCTTATCGT
AR3077-arcA p2 RT qpcr	TCACGCGCTAACAGAAGACC
**Junction PCR**	
TM2872- Ins17 rev (P17)	GTTGGGACTGACGTTGCCGGTAATCG
TM2873- Ins18 for (P18)	CGGAAATGATTACTTATCTCTCGGTTACTACG
TM2874- Ins16 rev (P16)	CTCTTCAGAGACTGCCACATTAGCG
TM2862- Ins18 upcheck (P18’)	CACACAAACTCAGACATACGGTAACGG
AR2883 - pykA upcheck	ACGCGAAAACGACGCCATGGTGATTG
AR2884 - pykA downcheck	GCGGCGGATGAATGAAGAAGTCGAG
**pCL25 – *arn* op**	
MM2864-arn op seva2351 fwd	TATTTGATGCCTTTAAACCGTAATACCTGTTTTATCTGAC
MM2908-arnop seva2350 rev2	ACGCGGCCGCAAGCTGATCTTAGTGAATGGGTGAAAGA
TM2929- pCL25 rev	AGCTTGCGGCCGCGTCCAGATCTGGATCTGG
TM2930-pCL25 for	TAAAGGCATCAAATAGAATTCCCGACAGTAAG

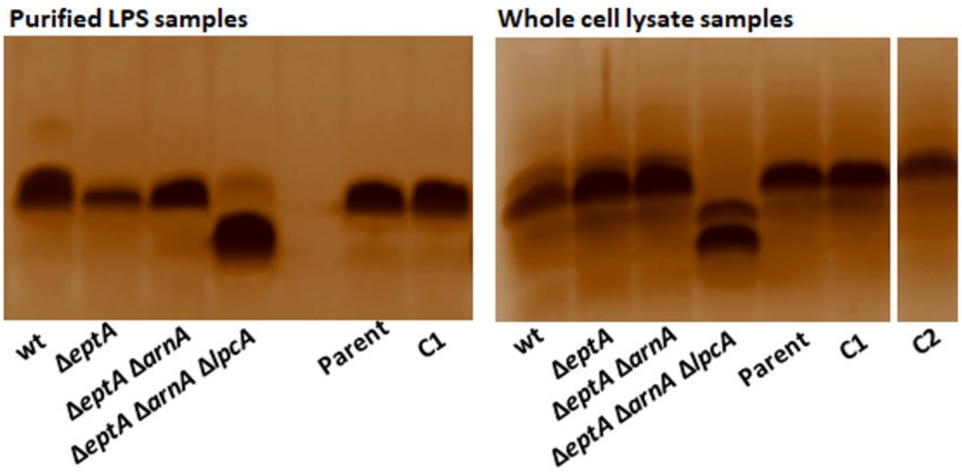

Figure S1: SDS-PAGE analysis of LPS extracted from PMB-resistant isolates. Silver stained profiles of purified LPS or from whole cell lysates. *E. coli* BL21(DE3) strains are used as controls where wt, Δ*eptA*, and Δ*eptA*Δ*arnA* contain the full core but variable lipid A modification and Δ*eptA*Δ*arn*Δ*lpcA* is a truncated (Re-LPS) chemotype without modification.

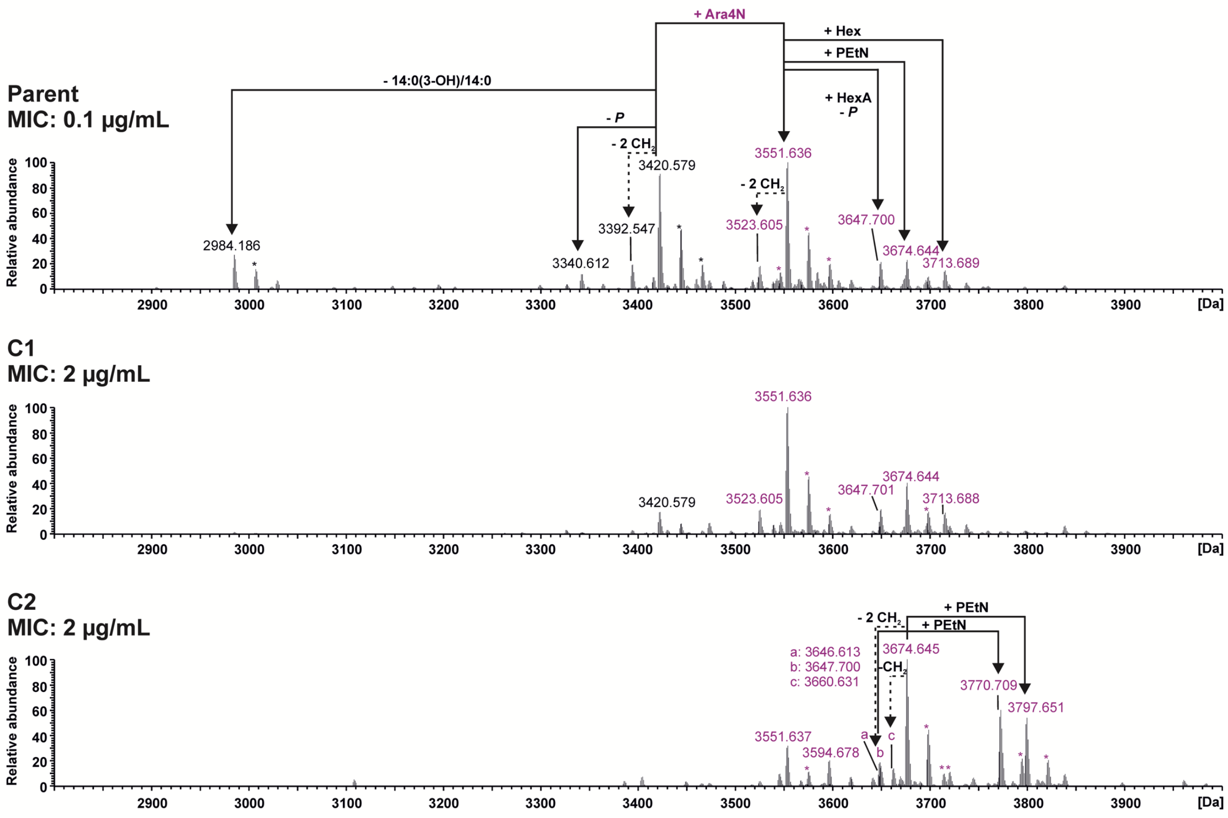

Figure S2: Polymyxin B-resistant isolates C1/C2 have increased levels of Ara4N modification on LPS. ESI-MS spectra of LPS isolated from the PMB-sensitive Parent strain (*top*) and PMB-resistant isolates C1 (*middle*) and C2 (*bottom*). Charge-deconvoluted spectra of the MS analyses performed in negative ion mode are depicted (section: 2800–4000 Da). All Ara4N-containing LPS species are labeled in *pink*, while all non-Ara4N containing LPS species are in *black*. Non-stoichiometric substitutions of the oligosaccharide core with PEtN, hexose (Hex), hexuronic acid (HexA) and phosphate (*P*) are indicated. Variations in total acyl chain length (-CH_2) and sodium ion adducts (*) are indicated. Calculated monoisotopic masses for observed LPS species are summarized in Table 1 in main text. Spectra of the other biological replicates are shown in main text (Fig. 2A).

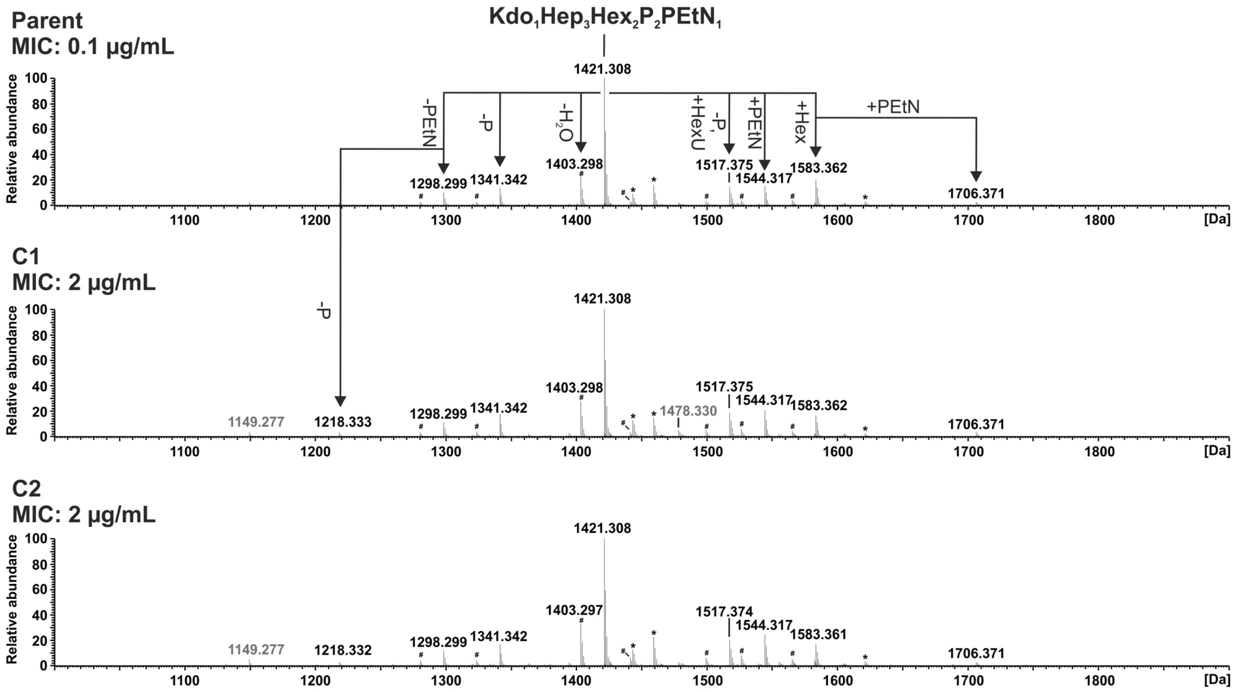

Figure S3: Mass spectrometric analysis does not indicate the presence of Ara4N-substituents on core oligosaccharides in either the PMB-sensitive Parent strain or the derived PMB-resistant isolates. Charge-deconvoluted spectra of MS analyses of core oligosaccharides – released from LPS (see Fig. 2A for MS spectra of the respective LPS) by 0.5-hour mild acidic hydrolysis – performed in negative ion mode are depicted (section: 1000–1900 Da). The most intensive peak at 1421.308 Da corresponds to the molecule $Kdo_1Hep_3Hex_2P_2PEtN_1$ (calculated monoisotopic mass: 1421.306 Da), consistent with previously assignments for the core oligosaccharide from *E. coli* B strains (3). Note: the second Kdo is cleaved during hydrolysis. Sodium (Δm = +21.98 Da) and potassium (Δm = +37.95 Da) ion adducts (*) and anhydro-compounds (Δm = –18.01 Da) (#) are indicated. Peaks with grey labels result from molecules with unassigned structures.

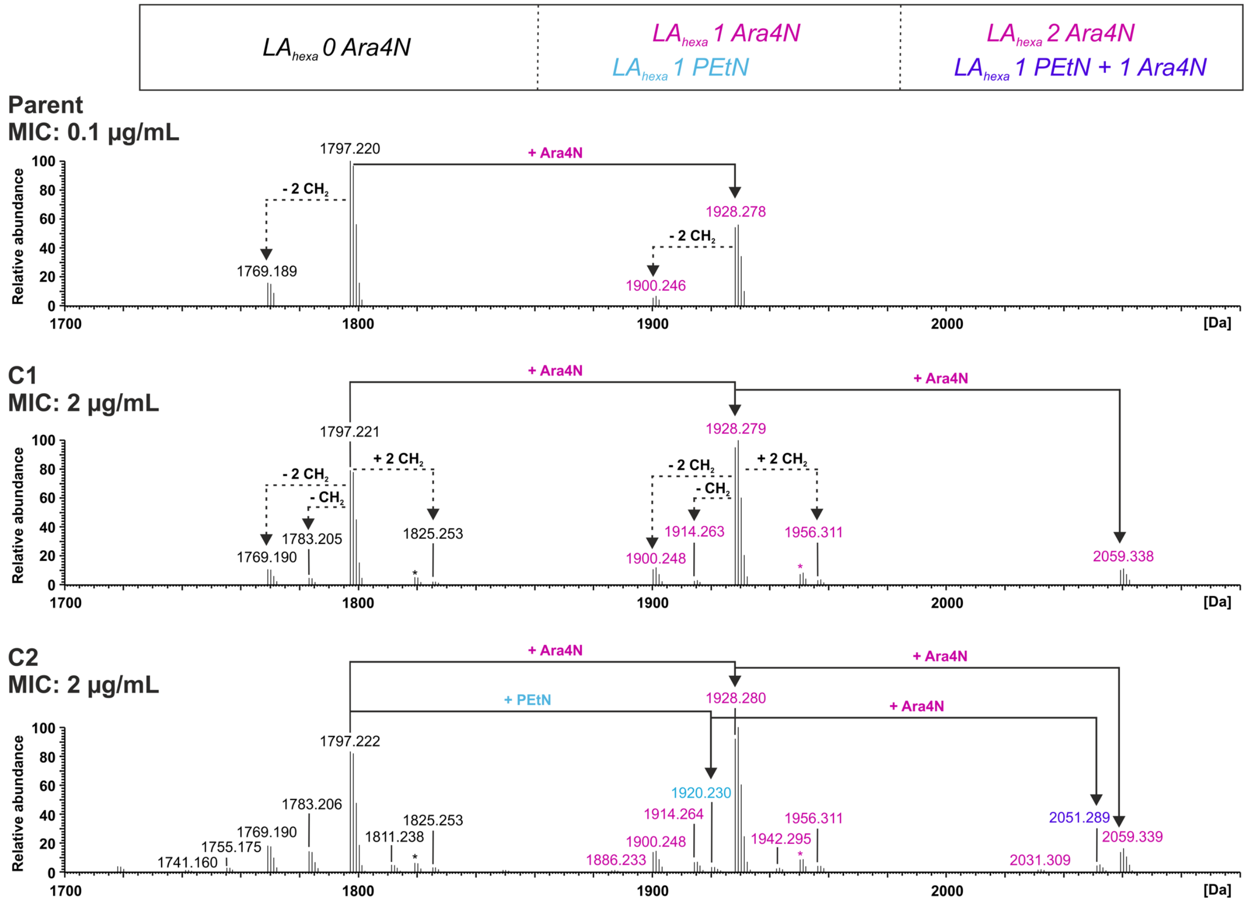

Figure S4: Mass spectrometric analysis confirms increased Ara4N-substitution on lipid A in PMB-resistant C1/C2 isolates. The charge-deconvoluted spectra of MS analyses of lipid A released from LPS (see Fig. 2A for MS spectra of the respective LPS) by 0.5-hour mild acidic hydrolysis as was analyzed in negative ion mode (section: 1700–2100 Da). Ara4N-substituted lipid A species are labeled in *pink*. In C2, a small population (~2%) of PEtN-containing species [single PEtN (*light blue*) or PEtN/Ara4N double modification (*violet*)] presumably arising from the promiscuous activity of other LPS PEtN transferase (EptB/C) was also observed. All unmodified lipid A species are in *black*. LA_{hexa} = lipid A, hexa-acylated: 2*GlcN, 2 P, 2*14:0(3-OH), 1*14:0[3-O(12:0)], 1*14:0[3-O(14:0); calculated monoisotopic mass: 1797.219 Da; LA_{hexa} + 1 PEtN: 1920.228 Da; LA_{hexa} + 1 Ara4N: 1928.278 Da; LA_{hexa} + 2 Ara4N: 2059.336 Da. Differences with a multiple of ±14.015 Da represent species with altered overall fatty acid chain length, sodium (Δm = +21.98 Da) ion adducts (*) are indicated.

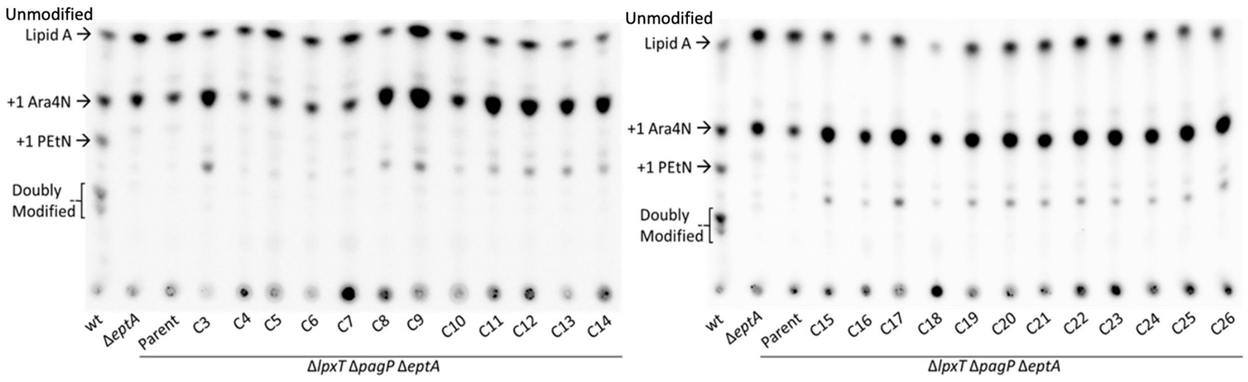

Figure S5: PMB-resistant isolates from the Parent background have increased relative Ara4N-lipid A modification levels. TLC analysis of ^{32}P-labeled lipid A isolated from *E. coli* BL21(DE3) wt, Δ*eptA,* Parent, and independently derived C3-C26 PMB-resistant isolates. Signals corresponding to lipid A alone or with modification are indicated, with assignments based on migration R_f comparison to standards.

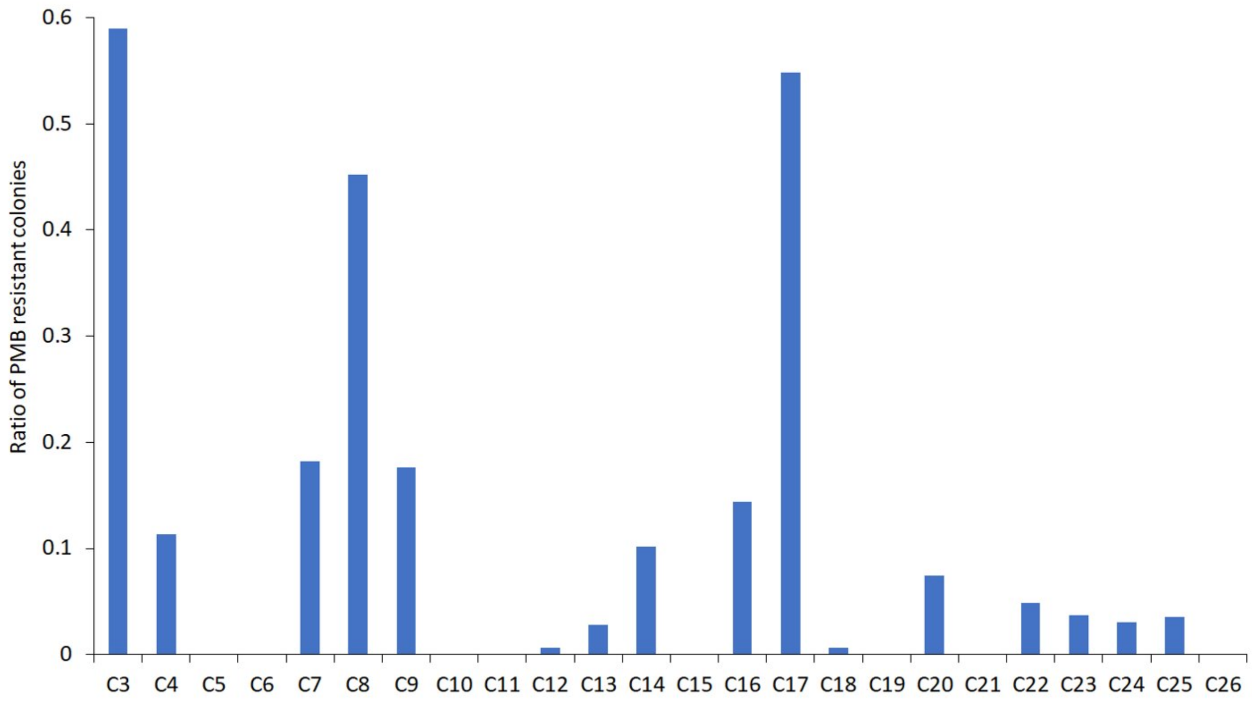

Figure S6: PMB-resistant isolates revert to the PMB-sensitive phenotype in the absence of PMB. The ratio of PMB-resistant to total colonies obtained after passaging isolates C3-C26 for 10 days in LB media lacking PMB.

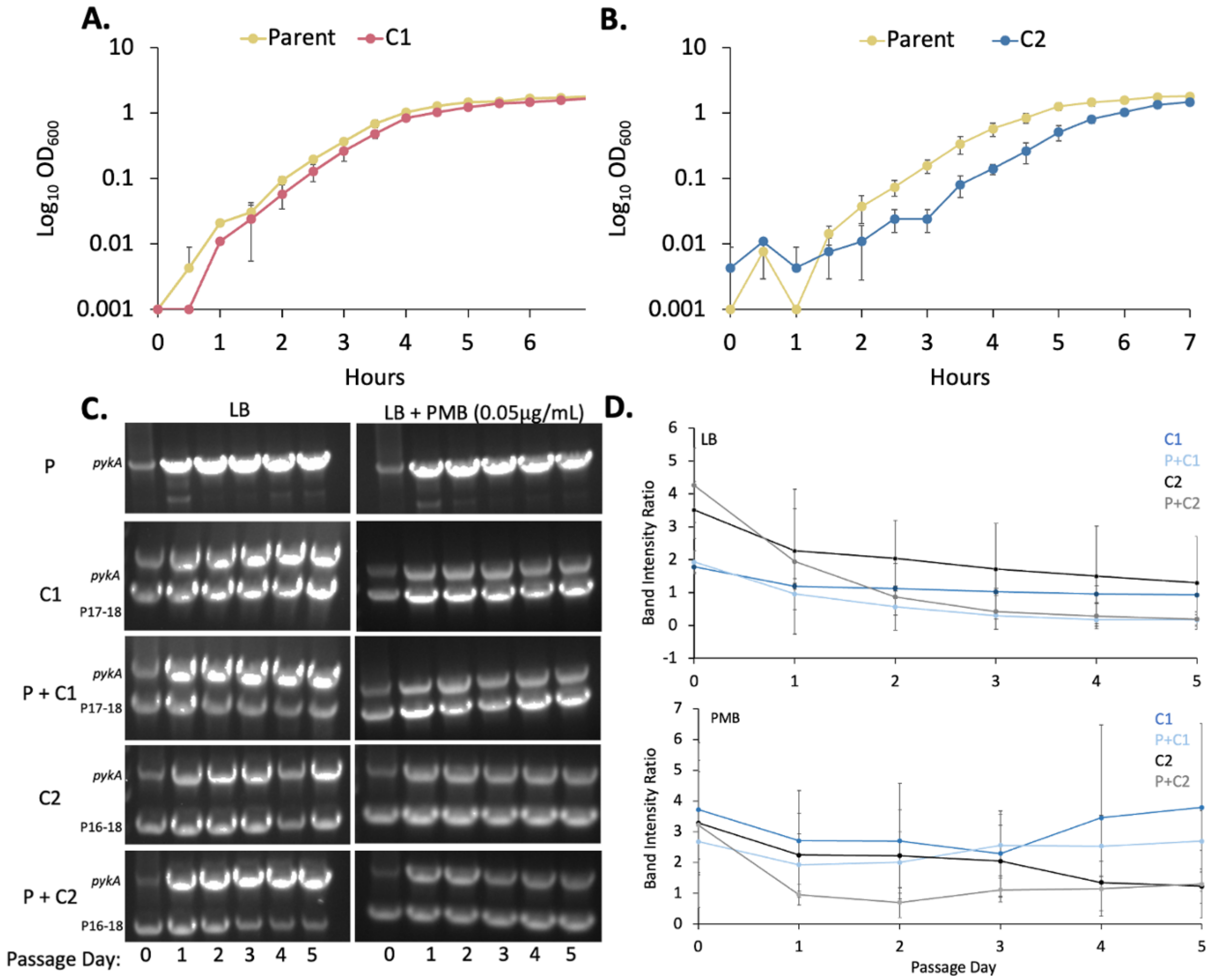

Figure S7: C1 and C2 IS*1*-18 dependent chromosomal amplifications impart minimal fitness cost. Growth curves of the Parent strain versus C1 (panel **A**) and C2 (panel **B**) in LB are plotted on log scale, with each fit representing the average of three biological replicates with error bars indicating standard deviation. **C.** Junction check multiplex PCRs performed on mixed and monocultures of Parent with C1 and C2 passaged in LB and LB supplemented with sub-MIC concentrations of PMB (0.05 µg/mL) for 5 days show modest fitness advantage of Parent over the mutants. Data is representative of 3 independent experiments (*n* = 3 biological replicates for each condition) **D.** Average band intensity ratios of Junction band (P17-18 in C1 and P16-18 in C2) to control band (*pykA*) from C, quantified using ImageJ. Error bars represent standard deviation within three biological replicates per condition (n = 3 biological replicates per condition).

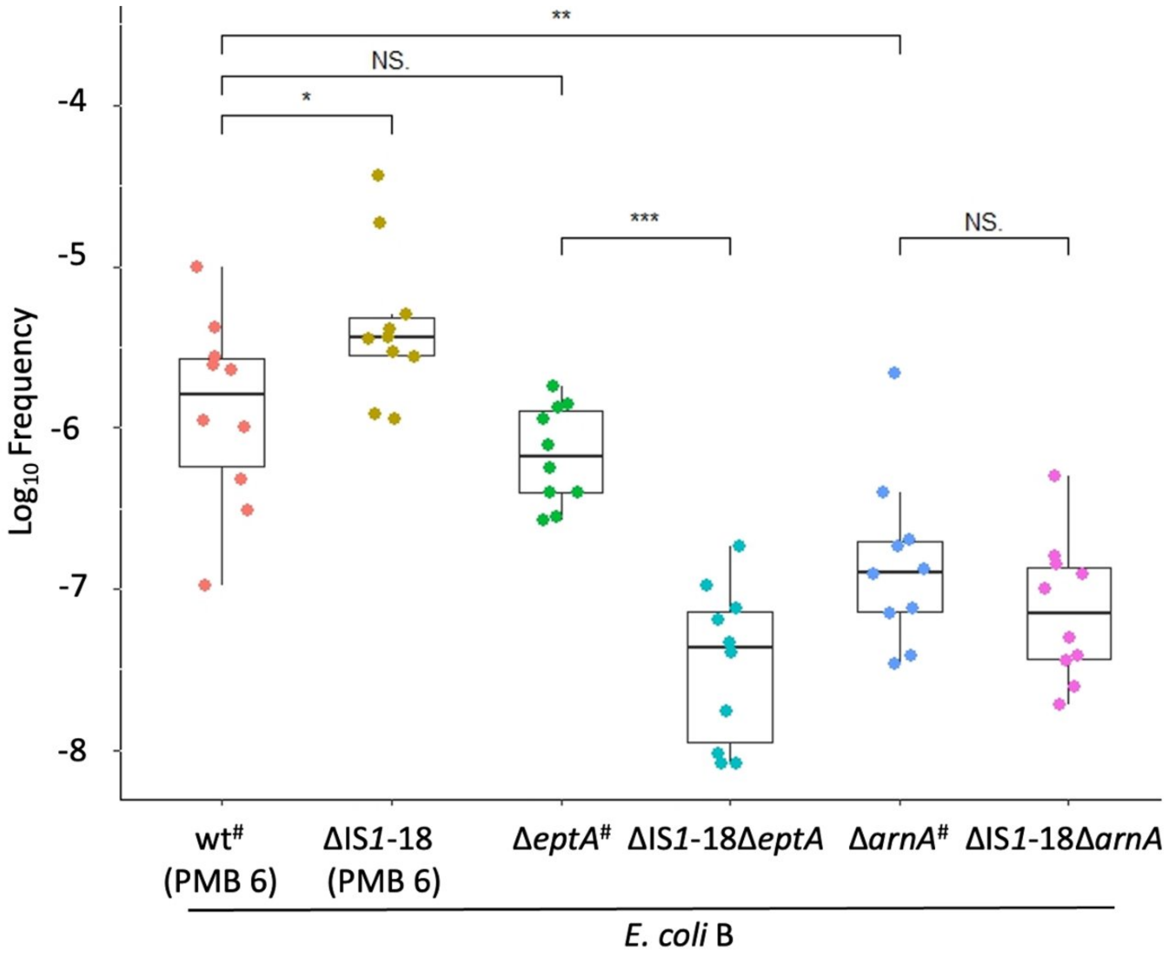

Figure S8: IS*1*-18 is required for high frequency PMB resistance in *arn*[+] strains lacking *eptA*. IS*1*-18 was deleted in *E. coli* BL21 (DE3) wt, ∆*eptA*, and ∆*arnA* backgrounds and the frequency of resistance was determined from 10 separate cultures per strain by plating dilutions of exponential growths (OD_{600nm} of 0.2 to 0.7) on LBA only or with 1 µg/mL of PMB (6 µg/mL for wt and ∆IS*1*-18, PMB 6) to calculate the ratio of PMB-resistant CFU. Significance values are based on Student's t-test (*p< 0.05, **p< 0.01, ***p <0.001, NS not significant). #- FOR data re-plotted from Figure 1 in main text for direct comparison.

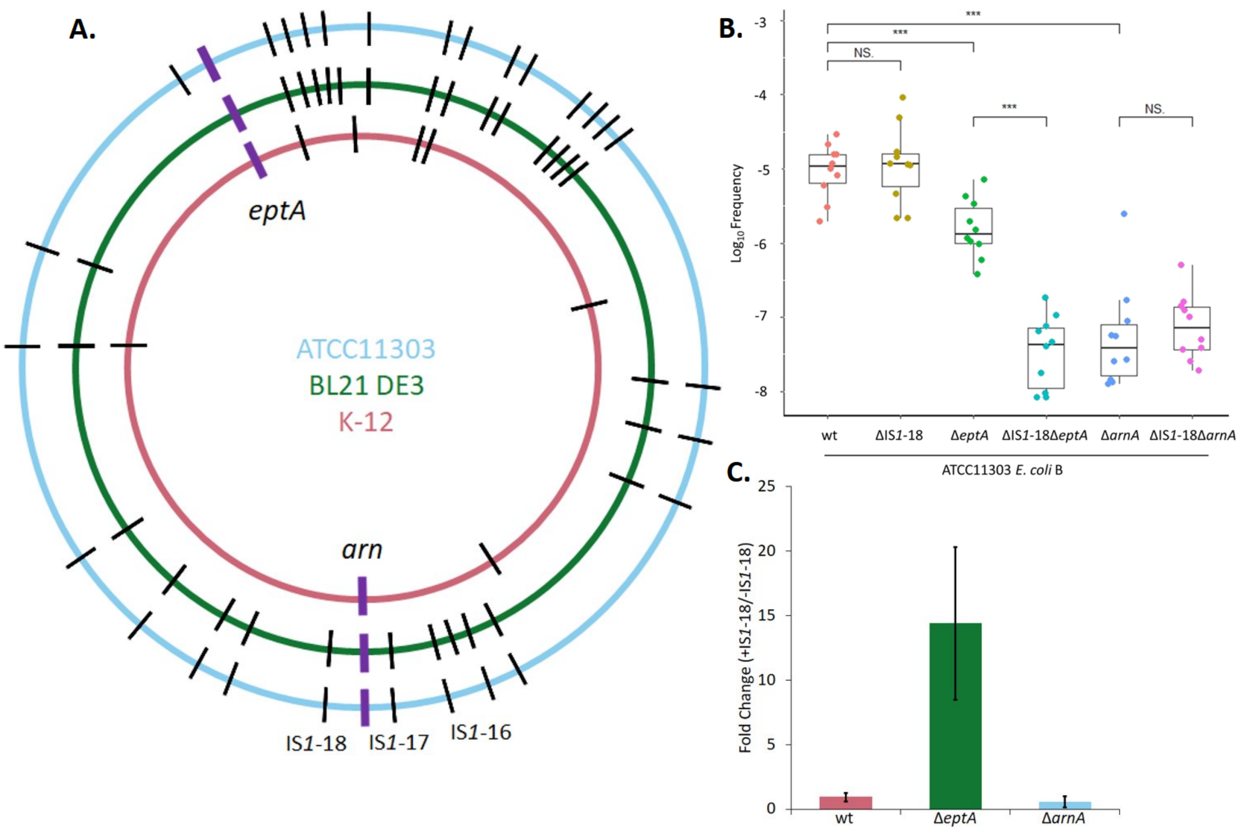

Figure S9: IS*1* distribution dictates PMB heteroresistance in wildtype *E. coli* B ATC11303 lineage. **A.** Relative positioning of IS*1* elements across the genome of *E. coli* B ATCC11303 (light blue), *E. coli* BL21(DE3) (green), and the *E. coli* BW25113 K-12 strain (pink) with 28, 29, and 7 IS*1* elements respectively (black dashes). The relative locations of *eptA* and the *arn* operon are indicated by thick purple dashes. The positions of IS*1*-16, IS*1*-17, and IS*1*-18 are indicated, and is shared between both *E. coli* B strains but not the K-12 strain. **B.** In the *E. coli* B ATC11303 background frequency of resistance (FOR) was determined for wt, Δ*eptA*, and Δ*arnA* each with and without IS*1*-18. The FOR was determined from 10 separate cultures per strain by plating dilutions of exponential growing cells (OD_{600nm} 0.2 to 0.7) on LBA containing 1 µg/mL PMB (8 µg/mL for wt and ΔIS*1*-18), then measuring the ratio of PMB-resistant CFUs to total plated cells after overnight incubation. Significance values based on Student's t-test (***p <0.001, NS not significant). **C.** Fold change in median FOR when deleting *IS1*-18 was quantified for *E. coli* B ATC11303 wt, Δ*eptA*, and Δ*arnA* with fold changes of approximately 1, 14, and 1 respectively. Error bars were derived from propagating median absolute deviation of values in the interquartile range.

References

1. Komazin G, Maybin M, Woodard RW, Scior T, Schwudke D, Schombel U, Gisch N, Mamat U, Meredith TC. 2019. Substrate structure-activity relationship reveals a limited lipopolysaccharide chemotype range for intestinal alkaline phosphatase. J Biol Chem 294:19405-19423.
2. Datsenko KA, Wanner BL. 2000. One-step inactivation of chromosomal genes in *Escherichia coli* K-12 using PCR products. Proc Natl Acad Sci U S A 97:6640-5.
3. Klein G, Muller-Loennies S, Lindner B, Kobylak N, Brade H, Raina S. 2013. Molecular and structural basis of inner core lipopolysaccharide alterations in *Escherichia coli*: incorporation of glucuronic acid and phosphoethanolamine in the heptose region. J Biol Chem 288:8111-8127.
